# Risk-Guided Screening for Atrial Fibrillation Using Electronic Health Records

**DOI:** 10.1161/CIRCULATIONAHA.126.079391

**Published:** 2026-07-28

**Authors:** Ramesh Nadarajah, Jianhua Wu, Ali Wahab, Catherine Reynolds, Mohammad Haris, Tobin Joseph, Keerthenan Raveendra, Ben Hurdus, Khalid Kazi, Sheena Bennett, Chris Hayward, Ben Mercer, Jing Kang, Chenyi Gao, Yoko M. Nakao, Koji Kawakami, Carlin Chang, Abraham Wai, Jiandong Zhou, Gary Tse, Talish Razi Benita, Lior Rokach, Ronen Arbel, Moti Haim, Doron Zahger, Dina Labib, Jacqueline Flewitt, James A. White, Konsta Teppo, Mika Lehto, Ville Langén, Aleksi K. Winstén, K.E. Juhani Airaksinen, Jari Haukka, Olli Halminen, Jukka Putaala, Juha Hartikainen, Miika Linna, Ben Freedman, Emma Svennberg, A. John Camm, Gregory Y.H. Lip, Chris P. Gale

**Affiliations:** 1 Leeds Institute of Data Analytics (R.N., A.W., C.R., M.H., T.J., K.R., B.H., K. Kazi, S.B., C.H., C.P.G.), University of Leeds 4468 4468 , Leeds, United Kingdom.; 2 Leeds Institute for Cardiovascular and Metabolic Medicine (R.N., A.W., C.R., M.H., T.J., B.H., K. Kazi, S.B., C.H., C.P.G.), University of Leeds 4468 4468 , Leeds, United Kingdom.; 3 Department of Cardiology, Leeds Teaching Hospitals NHS Trust 4472 , Leeds, United Kingdom (R.N., B.M., C.P.G.).; 4 Wolfson Institute of Population Health, Queen Mary, University of London, London, United Kingdom (J.W., C.G.).; 5 Oral Clinical Research Unit, Faculty of Dentistry Oral Craniofacial Sciences, King’s College London, London, United Kingdom (J.K.).; 6 Department of Pharmacoepidemiology, University of Kyoto, Kyoto, Japan (Y.M.N., K. Kawakami).; 7 Departments of Medicine (C.C.), University of Hong Kong, Hong Kong SAR, China.; 8 Emergency Medicine (A.W.), University of Hong Kong, Hong Kong SAR, China.; 9 Family Medicine and Primary Care (J.Z.), University of Hong Kong, Hong Kong SAR, China.; 10 Pharmacology and Pharmacy (J.Z.), University of Hong Kong, Hong Kong SAR, China.; 11 School of Public Health (J.Z.), University of Hong Kong, Hong Kong SAR, China.; 12 Department of Cardiology, Tianjin Institute of Cardiology, Second Hospital of Tianjin Medical University, Tianjin, China (G.T.).; 13 School of Nursing and Health Sciences, Hong Kong Metropolitan University, Hong Kong SAR, China (G.T.).; 14 Community Medical Services Division, Clalit Healthcare Services, Tel Aviv, Israel (T.R.B., R.A., M.H., D.Z.).; 15 Department of Information Systems and Software Engineering, Ben Gurion University of the Negev, Beer-Sheva, Israel (T.R.B., L.R.).; 16 Maximizing Health Outcomes Research Lab, Sapir College, Sderot, Israel (R.A.).; 17 Department of Cardiology, Soroka University Medical Center, Beer Sheva, Israel (M.H., D.Z.).; 18 Department of Cardiac Sciences and Libin Cardiovascular Institute, Cumming School of Medicine, University of Calgary, Calgary, AB, Canada (D.L., J.F., J.A.W.).; 19 Department of Cardiovascular Medicine, Cairo University, Cairo, Egypt (D.L.).; 20 Heart Center (K.T., K.E.J.A.), Turku University Hospital and University of Turku, Turku, Finland.; 21 Division of Medicine (V.L.), Turku University Hospital and University of Turku, Turku, Finland.; 22 Jorvi Hospital, Department of Internal Medicine (M. Lehto), HUS Helsinki University Hospital and University of Helsinki, Helsinki, Finland.; 23 Department of Neurology (J.P.), HUS Helsinki University Hospital and University of Helsinki, Helsinki, Finland.; 24 Department of Mathematics and Statistics, University of Turku 8058 , Turku, Finland (A.K.W.).; 25 Department of Public Health, University of Helsinki, Helsinki, Finland (J. Haukka).; 26 Departments of Health and Social Management (O.H.), University of Eastern Finland, Finland.; 27 Health and Social Management (M. Linna), University of Eastern Finland, Finland.; 28 Heart Center, Kuopio University Hospital and University of Eastern Finland (J. Hartikainen).; 29 Department of Industrial Engineering and Management, Aalto University, Espoo, Finland (M. Linna).; 30 Heart Research Institute, Sydney Medical School, Charles Perkins Center, and Cardiology Department, The University of Sydney, Sydney, NSW, Australia (B.F.).; 31 Karolinska Institutet, Department of Medicine, Karolinska University Hospital, Stockholm, Sweden (E.S.).; 32 Molecular and Clinical Sciences Research Institute, City St George’s University of London, London, United Kingdom (A.J.C.).; 33 Institute of Life Course and Medical Sciences, University of Liverpool, Liverpool, United Kingdom (G.Y.H.L.).; 34 Department of Clinical Medicine, Aalborg University, Aalborg, Denmark (G.Y.H.L.).; 35 Department of Cardiology, Lipidology, and Internal Medicine with Intensive Coronary Care Unit, Medical University of Bialystok, Bialystok, Poland (G.Y.H.L.).

**Keywords:** atrial fibrillation, electronic health records, public health, screening

## Abstract

**BACKGROUND:**

Screening for atrial fibrillation (AF) on the basis of AF risk may be more effective. We aimed to develop, externally validate, and prospectively test a machine learning prediction model using electronic health records (EHRs) to guide AF screening.

**METHODS:**

We developed and validated a random forest prediction model for new AF within 6 months, using age, sex, and 10 comorbidities (Future Innovations in Novel Detection of Atrial Fibrillation [FIND-AF] 2.0) in EHRs in the United Kingdom (n=2 081 139), Japan (n=7 795 244), Israel (n=2 166 795), Canada (n=627 919), and China (n=149 145). We conducted a prospective study where participants ≥30 years old without AF and with a CHA_2_DS_2_-VASc score ≥2 in men and ≥3 in women, stratified by FIND-AF 2.0 into high and low risk, undertook 4 ECG recordings per day for 3 weeks using a handheld ECG recorder, with a primary outcome of newly diagnosed AF. We estimated stroke risk associated with nonanticoagulated AF in patients with high FIND-AF 2.0 risk in the FinACAF (Finnish Anticoagulation in Atrial Fibrillation) registry of patients with AF (n=229 565).

**RESULTS:**

FIND-AF 2.0 was applicable to all EHRs and showed good to excellent prediction performance (United Kingdom: area under the receiver operating characteristic curve [AUROC], 0.819 [95% CI, 0.809–0.829]; Israel: AUROC, 0.835 [95% CI, 0.828–0.842]; Japan: AUROC, 0.751 [95% CI, 0.745–0.757]; Canada: AUROC, 0.747 [95% CI, 0.741–0.753]; China: AUROC, 0.753 [95% CI, 0.725–0.771]), with AUROC>0.7 in men and women in all cohorts, and improved performance compared with CHA_2_DS_2_-VASc and C_2_HEST (coronary artery disease or chronic obstructive pulmonary disease [1 point each]; hypertension [1 point]; elderly [age ≥75 years, 2 points]; systolic HF [2 points]; thyroid disease [hyperthyroidism, 1 point]). Of 1923 participants from 15 sites in the prospective study (mean age, 70.2 [SD 9.4] years), with a mean of 74.8 (SD, 19.4) ECG recordings, AF was diagnosed in 5 of 902 (0.6%) with low FIND-AF 2.0 risk and 46 of 1021 (4.5%) with high FIND-AF 2.0 risk (odds ratio, 8.46 [95% CI, 3.35–21.40], *P*<0.001). Median AF burden among high FIND-AF 2.0 risk–detected cases was 33.4% (interquartile range, 5.1%–91.6%), and 96.1% initiated oral anticoagulants. In the FinACAF registry, the rate of ischemic stroke for patients with high FIND-AF 2.0 risk, AF, and no anticoagulants was 6.0 events per 100 patient-years.

**CONCLUSIONS:**

The EHR-based machine learning model, FIND-AF 2.0, identifies a high-risk subpopulation for AF diagnosis among patients at elevated risk of stroke and could enable scalable, EHR-driven, risk-guided AF screening.

**Clinical Perspective**

**What Is New?**

- A machine learning prediction model (Future Innovations in Novel Detection of Atrial Fibrillation 2.0), validated in 13 million individuals across 5 countries, could be implemented in nationwide electronic health records to accurately predict incident atrial fibrillation.
- In a prospective atrial fibrillation screening study, high Future Innovations in Novel Detection of Atrial Fibrillation 2.0 risk was associated with increased detection of atrial fibrillation among those at elevated risk of stroke.

**What Are the Clinical Implications?**

- Atrial fibrillation screening can be more accurately guided at scale using the Future Innovations in Novel Detection of Atrial Fibrillation 2.0 model in nationwide electronic health records to increase detection of undiagnosed atrial fibrillation.
- Randomized evidence is required to assess whether targeted atrial fibrillation screening with Future Innovations in Novel Detection of Atrial Fibrillation 2.0 improves clinical outcomes.

Atrial fibrillation (AF) is a common cause of stroke but underdiagnosed in routine care.^1,2^ Screening older individuals or those with elevated CHA_2_DS_2_-VASc score with ECG monitoring is associated with increased AF detection compared with routine care.^3^ While advancing age and the variables in the CHA_2_DS_2_-VASc score are associated with increased AF incidence,^4,5^ it may be possible to more precisely identify high-risk individuals for AF screening. Electronic health records (EHRs) and insurance databases are increasingly available at national scale across many countries,^6^ and a multivariable prediction model using these data to calculate risk of newly diagnosed AF could offer an internationally scalable solution to better guide AF screening.

Previous models developed or validated to predict risk of newly diagnosed AF in EHRs have shortcomings,^7^ including long prediction horizons that identify AF far in the future and the requirement for variables frequently missing from routinely collected data, which limits the scale of their application (Supplementary Material). Furthermore, they have seldom been prospectively tested to see whether they can effectively guide AF screening to increase AF detection. In 2022, a machine learning prediction model (Future Innovations in Novel Detection of Atrial Fibrillation [FIND-AF]) was developed using United Kingdom primary care EHRs. It had excellent prediction accuracy and was robust to missing data in routine care^8^ but required 75 variables, which limited its implementation.

To address this, we developed a parsimonious version of the FIND-AF machine learning model (FIND-AF 2.0) in United Kingdom primary care, externally validated it in 4 countries (Japan, Israel, Canada, and China), and conducted a prospective multicenter interventional nonrandomized validation study to determine whether FIND-AF 2.0 provides additional risk stratification beyond previous AF screening strategies and whether AF detected with FIND-AF 2.0 risk guided screening is actionable in terms of AF burden and initiation of oral anticoagulation treatment.

## METHODS

Data from the retrospective analyses are not publicly available and can be acquired from third parties. Data from the prospective study are available from the corresponding author upon reasonable request. To support further external validation studies, the R code used to derive and evaluate the FIND-AF 2.0 model is available to other researchers and health systems upon request to the corresponding author.

### Study Design

This research program sought to develop, externally validate, and prospectively validate a parsimonious machine learning model (FIND-AF 2.0) to guide AF screening. The protocols have been described previously.^9,10^ We followed the Transparent Reporting of a Multivariable Prediction Model for Individual Prognosis and Diagnosis and Artificial Intelligence statement,^11^ with full details of the methods for each component of this research program available in the Supplemental Methods.

### Development, Internal Validation, and Multinational External Validation of FIND-AF 2.0

We included individuals ≥30 years old without a prior diagnosis of AF or atrial flutter from Clinical Practice Research Datalink-GOLD (United Kingdom), the Japan Medical Insurance Claims Database (Japan), Clalit Health Service (Israel), the Cardiovascular Imaging Registry of Calgary (Canada), and the Clinical Data Analysis and Reporting System (Hong Kong SAR, China). Individuals were assigned a diagnosis of AF or atrial flutter, death, withdrawal from the database, or 6 months, whichever came first.

The outcome was newly diagnosed AF or atrial flutter within the next 6 months based on diagnostic code lists. A final selection of 12 predictor variables was selected through a consensus process to include age (as a continuous variable), sex, and the following comorbidities: diabetes, heart failure, hypertension, stroke or transient ischemic attack, ischemic heart disease, chronic obstructive pulmonary disease, valvular heart disease, chronic kidney disease, rheumatoid arthritis, and hyperthyroidism (Figure S1).

To develop FIND-AF 2.0, the United Kingdom cohort was split into development (80%) and internal validation datasets (20%) using the Mersenne twister pseudorandom number generator. A random forest classifier (FIND-AF 2.0) was trained over the development cohort using 10-fold cross-validation for hyperparameter tuning (Supplemental Methods). To address class imbalance due to low AF incidence within 6 months, we applied class weighting during random forest model training. Variable importance was quantified using the Gini impurity measure, which is a measure of how each variable contributes to the homogeneity of nodes and leaves in the resulting random forest.

The model was compared with the original FIND-AF model, CHA_2_DS_2_-VASc, and C_2_HEST scores (coronary artery disease or chronic obstructive pulmonary disease [1 point each]; hypertension [1 point]; elderly [age ≥75 years, 2 points]; systolic HF [2 points]; thyroid disease [hyperthyroidism, 1 point]) in the United Kingdom validation dataset and then against CHA_2_DS_2_-VASc and C_2_HEST in the Japan, Israel, Canada, and China datasets. The CHA_2_DS_2_-VASc and C_2_HEST scores were chosen for comparison because they can be calculated at scale in routinely collected EHRs and were the best-performing models for AF prediction in EHRs in a systematic review and meta-analysis,^7^ and CHA_2_DS_2_-VASc score has been used to guide eligibility for AF screening trials.^3^ Other scores developed for AF risk prediction (such as CHARGE-AF [Cohorts for Heart and Aging Research in Genomic Epidemiology Model for Atrial Fibrillation])^12^ were not included because they require variables that are often missing in routine EHR data, limiting their scalability (Supplemental Methods).

### Prospective Interventional Clinical Validation of FIND-AF 2.0

We tested FIND-AF 2.0 in a single-arm interventional prospective validation study. Ethics approval was given by the North West – Greater Manchester South Research Ethics Committee (reference 23/NW/0180).

Patients ≥30 years old, with a primary care EHR, no prior history of AF or atrial flutter, not on the palliative care register or receiving long-term anticoagulation and indicated for oral anticoagulation if AF was detected (men with a CHA_2_DS_2_-VASc ≥2 and women with a CHA_2_DS_2_-VASc ≥3) were eligible. This approach was chosen to enroll patients who would be eligible for oral anticoagulants for stroke prevention if AF was detected^5^ and to determine whether FIND-AF 2.0 risk assessment provides additional risk stratification among individuals with elevated CHA_2_DS_2_-VASc score who constitute the population normally recruited into randomized controlled trials (RCTs) of AF screening.^3^

Patients were enrolled according to FIND-AF 2.0 risk categorization with invitation by text and post. Patients with a FIND-AF 2.0 risk score of ≥0.0053 were classified as high risk, and recruitment was made in batches with the aim that 55% of the prospective study population would be high risk.^10^ The FIND-AF 2.0 risk score of ≥0.0053 was chosen as the threshold to reflect the top decile of FIND-AF 2.0 risk in the general population without baseline AF, in accordance with previous studies of risk-guided AF screening.^13^

Consenting participants received a handheld single-lead ECG recorder (Zenicor II, Zenicor, Stockholm, Sweden) by post and were asked to record their ECG 4 times daily (morning, noon, afternoon, and evening), or whenever they had palpitations, for 3 weeks.^14^

The primary outcome was newly diagnosed AF during ECG monitoring. AF was diagnosed when there was at least 1 episode of completely irregular heart rhythm without P waves lasting 30 seconds,^15^ in accordance with previous RCTs using intermittent ECG monitoring for AF screening.^1,4^ All diagnoses of AF were confirmed by 2 electrophysiologists blinded to risk classification of the participant (B.M. and T.S.). Newly diagnosed AF was informed to the participant and their primary care physician, with follow-up care at the discretion of their primary care physician. The secondary outcome was prescription of oral anticoagulants to participants newly diagnosed with AF during ECG monitoring. Safety outcomes included other arrhythmias detected during ECG monitoring.

### Association of FIND-AF 2.0 Risk With Ischemic Stroke in Patients With AF Not Taking Anticoagulants

Given that the primary aim of AF screening is to prevent ischemic stroke through earlier detection and anticoagulation treatment of undiagnosed AF, we assessed the association of high FIND-AF 2.0 risk with ischemic stroke in patients ≥30 years old with AF who did not take anticoagulants in the FinACAF (Finnish Anticoagulation in AF, Finland) registry. Follow-up began at the time of the first AF diagnosis and continued until the first ischemic stroke event, the first oral anticoagulant purchase, death, or a maximum of 1 year after AF diagnosis, whichever occurred first.

### Statistical Analysis

For the development and validation of FIND-AF 2.0, discrimination was primarily assessed using area under the receiver operating characteristic curve (AUROC), which is less sensitive to outcome prevalence and therefore more suitable for comparison across external validation datasets with differing event rates. Given the rarity of the outcome, the area under the precision–recall curve was also reported as a complementary measure for each dataset. AUROC values of <0.60, 0.60 to 0.70, 0.70 to 0.80, and >0.80 were defined a priori as inadequate, adequate, good, and excellent on the basis of prior publications.^16^ Calibration was assessed by the calibration slope, and Brier score was calculated. We conducted subgroup analysis by sex with the DeLong test to compare AUROCs between men and women.

For the prospective clinical validation of FIND-AF 2.0, we calculated the odds ratio (OR) for AF detection during ECG monitoring between high and low FIND-AF 2.0 risk. Our power calculations estimated that, if the detection rate was 6% in the high-risk group^13,17^ and 1.5% in the low-risk group,^1,18^ 977 participants completing the study protocol (per protocol) in each of the high- and low-risk groups (1954 total) would provide 80% power at a 5% significance level to detect a significant difference.

We also calculated positive predictive value (PPV), negative predictive value (NPV), sensitivity, and F1 score at the prespecified threshold. AUROC and specificity were not assessed because the recruitment ratio of high to low FIND-AF 2.0 risk had been fixed in advance. We also explored sensitivity, PPV, NPV, and F1 score at different thresholds of FIND-AF 2.0, CHA_2_DS_2_-VASc, C_2_HEST, and CHARGE-AF scores and for the age-based approach (75 and 76 years) used in the STROKESTOP RCT.^1,19^ For the calculation of CHARGE-AF, we used a simplified calculation to enhance scalablity,^20^ and patients required data in their EHR for systolic blood pressure, weight, and smoking status within the 12 months before recruitment and height data at any point before recruitment.^21^ We also conducted an additional analysis adjusting NPV to a general population AF prevalence of 0.4%, as the prospective study selectively screened higher-risk individuals, as well as a subgroup analysis where subgroups had at least 5 AF events, including by age <75 years and ≥75 years, in men and women and in those with and without hypertension and diabetes.

To quantify the association of FIND-AF 2.0 risk with ischemic stroke in patients with AF but not taking anticoagulants in the FinACAF registry, stroke rates with 95% CI according to high- and low-risk FIND-AF 2.0 and high and low or intermediate CHA_2_DS_2_-VASc categorization were estimated with Poisson regression.

## RESULTS

### FIND-AF 2.0 Model Development and Internal and External Validation

In the development United Kingdom cohort, there were 2 081 139 individuals (1 664 911 in the training dataset, 416 228 in the testing dataset) with a mean age of 49.9 (SD, 15.4) years and 1 055 561 women (50.7%) (Table 1). Within 6 months, 7386 individuals (0.4%) were recorded as having AF (Table S1).

**Table 1.**
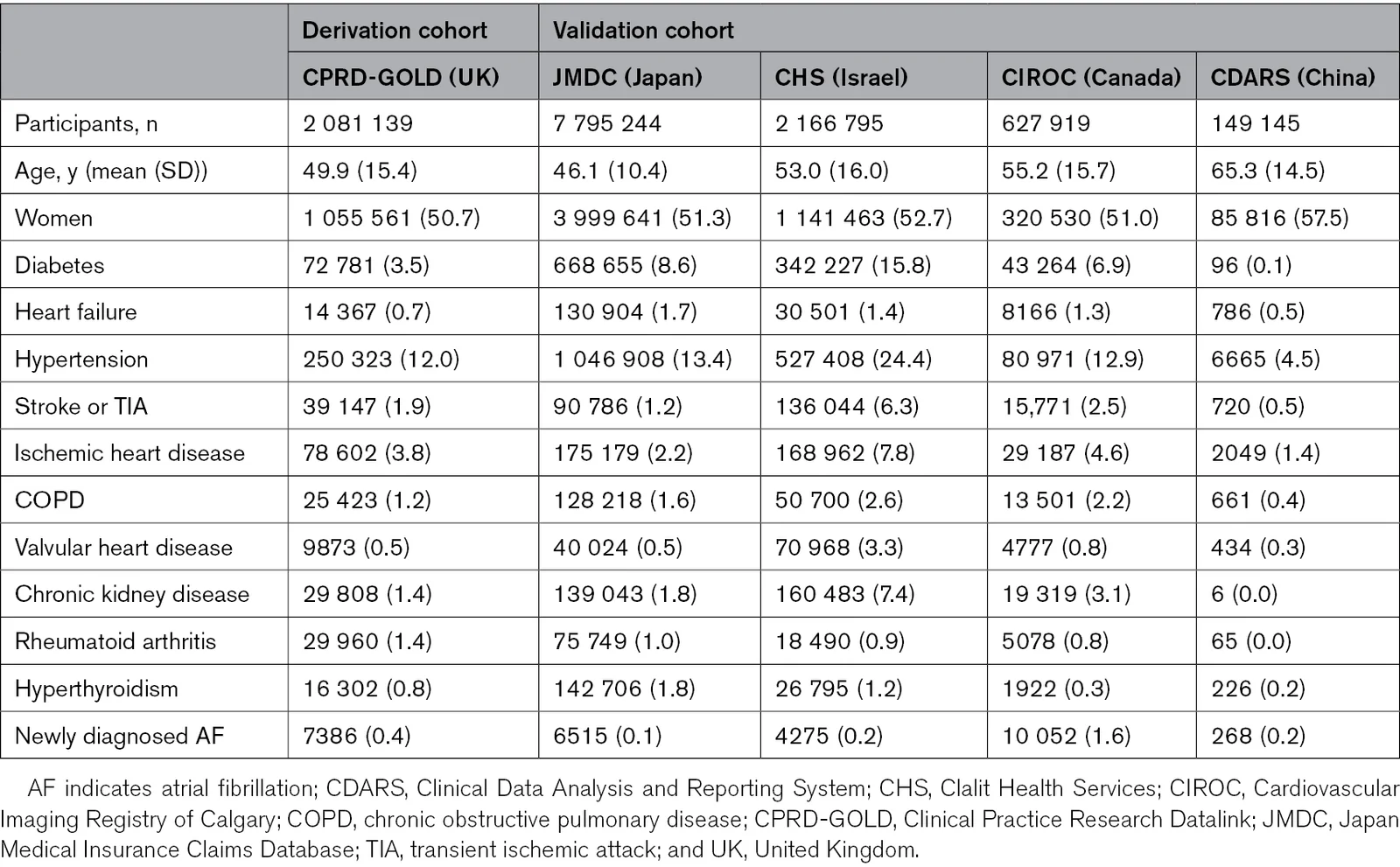
Characteristics of Patients in Derivation and External Validation Cohorts

**Figure 1.**
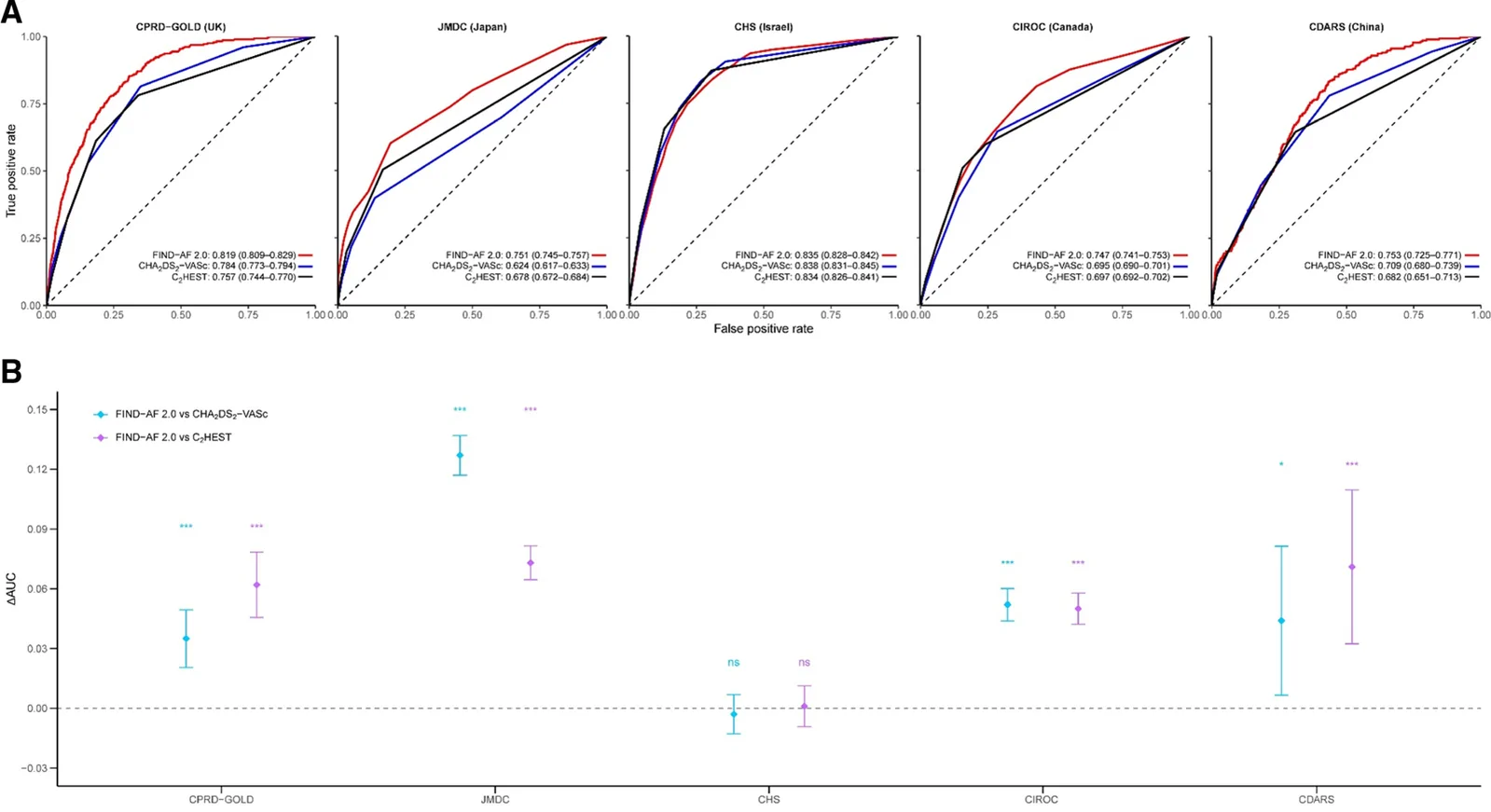
**Prediction performance for newly diagnosed atrial fibrillation (AF) within the next 6 months across the different cohorts for FIND-AF (Future Innovations in Novel Detection of Atrial Fibrillation) 2.0, CHA_2_DS_2_-VASc, and C_2_HEST. A**, Receiver operating characteristic curves comparing the discriminative performance of FIND-AF 2.0, CHA_2_DS_2_-VASc, and C_2_HEST for prediction of newly diagnosed AF within 6 months. **B**, Gap in the area under the receiver operating characteristic curve of FIND-AF 2.0 compared with CHA_2_DS_2_-VASc and C_2_HEST for prediction of newly diagnosed AF within 6 months. Error bars represent 95% CI. Δ indicates the difference between the models. ***P<0.001, **P<0.01, *P<0.05; ns, *P*≥0.05. AUC indicates area under the curve; C_2_HEST, coronary artery disease or chronic obstructive pulmonary disease (1 point each); hypertension (1 point); elderly (age ≥75 years, 2 points); systolic HF (2 points); thyroid disease (hyperthyroidism, 1 point); CHA_2_DS_2_-VASc, congestive heart failure, hypertension, age ≥75 years (doubled), diabetes mellitus, prior stroke or transient ischemic attack or thromboembolism (doubled), vascular disease, age 65 to 74 years, sex category; CDARS, Clinical Data Analysis and Reporting System; CHS, Clalit Health Services; CIROC, Cardiovascular Imaging Registry of Calgary; CPRD-GOLD, Clinical Practice Research Datalink; JMDC, Japan Medical Insurance Claims Database; ns, non-significant; and UK, United Kingdom.

On internal bootstrap validation in the holdout United Kingdom testing dataset, the prediction performance for FIND-AF 2.0 with 12 variables (AUROC, 0.819 [95% CI, 0.809–0.829]; calibration slope, 0.783 [95% CI, 0.744–0.825]; Brier score, 0.070) was not statistically significantly different from the original FIND-AF model that comprised 75 variables (AUROC, 0.824 [95% CI, 0.814–0.834]; calibration slope, 0.782 [95% CI, 0.743–0.824]; Brier score, 0.069; delta AUROC, 0.005 [95% CI, −0.009 to 0.019], *P=*0.49). The most important variables for prediction in FIND-AF 2.0 were, in descending order, age, heart failure, ischemic heart disease, valvular heart disease, and hypertension (Figure S2). AF discrimination was better when using FIND-AF 2.0 compared with CHA_2_DS_2_-VASc and C_2_HEST (Table 2; Figure 1; Figure S3) and was superior in sex-stratified analyses (Table S2).

**Table 2.**
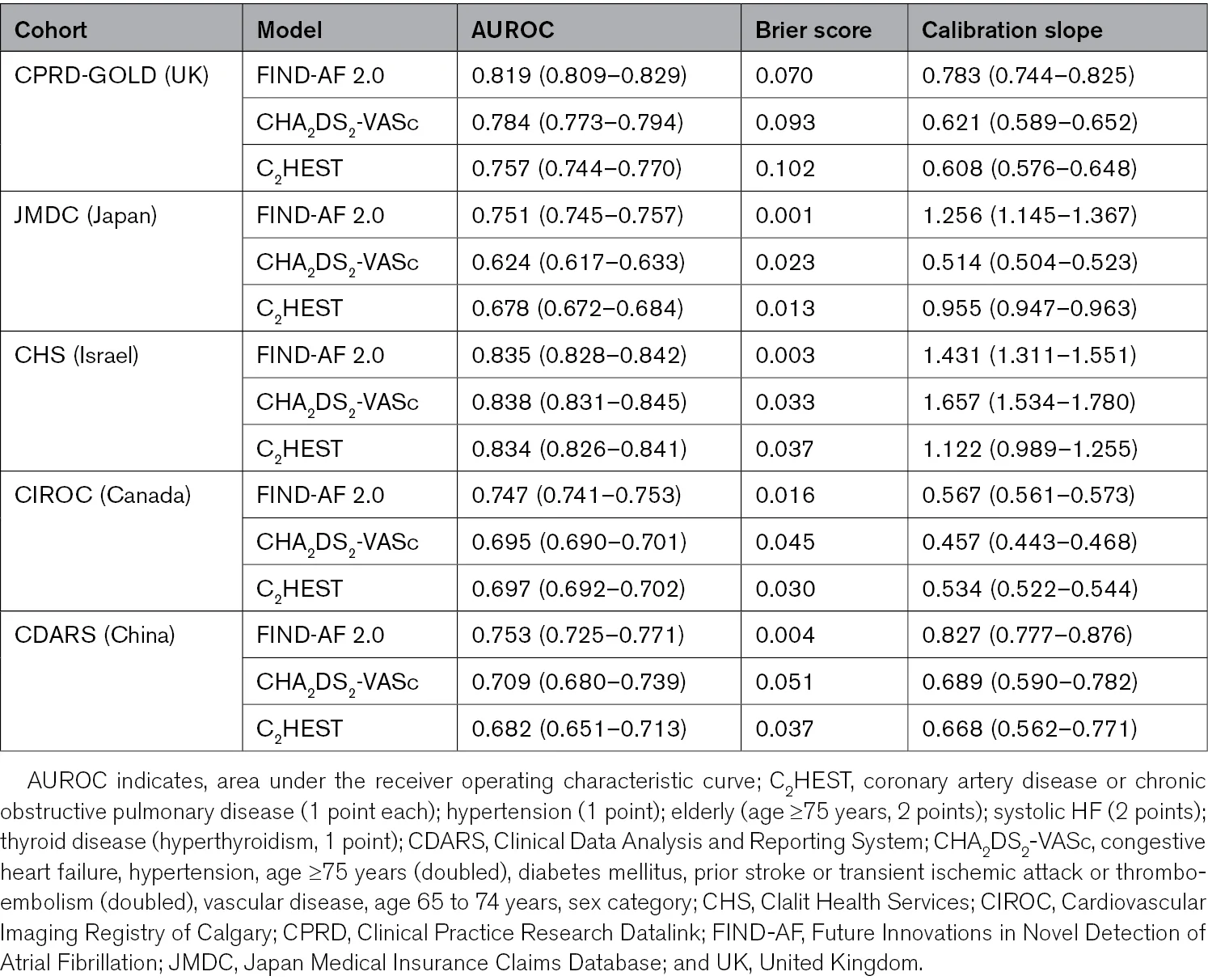
Prediction Performance of FIND-AF, CHA_2_DS_2_-VASc, and C_2_HEST for Newly Diagnosed Atrial Fibrillation Within 6 Months Across the Included Cohorts

The external validation sets comprised 7 795 244 patients from Japan, 2 166 795 patients from Israel, 627 919 patients from Canada, and 149 145 patients from China, with 6515 (0.1%), 4275 (0.2%), 10 052 (1.6%), and 268 (0.2%) AF cases at 6 months, respectively (Table 1; Table S1).

Across the external validation datasets, FIND-AF 2.0 could be applied to all records, with superior discrimination performance to CHA_2_DS_2_-VASc and C_2_HEST in Japan, Canada, and China (Table 2; Figure 1; Figure S3), while all models performed excellently in Israel with no statistically significant difference. This pattern was maintained in sex-stratified analyses, and FIND-AF 2.0 was the only model to demonstrate an AUROC ≥0.70 in both men and women across all international cohorts (Table S2). For the area under the precision–recall curve, FIND-AF 2.0 was superior compared with C_2_HEST and CHA_2_DS_2_-VASc in the United Kingdom, Japan, Canada, and China, with equal performance in Israel (Table S3). In each dataset, the area under the precision–recall curve for FIND-AF 2.0 was several times higher than the AF incidence rate, suggesting usefulness for rare event screening, including when stratified by sex (Table S3).

### Prospective Interventional Clinical Validation Study

Between October 4, 2023, and February 12, 2025, 127 216 patients at 15 primary care sites were screened for eligibility, and 17 063 were invited for participation. Of those invited, 1960 consented, with 1923 completing the intervention and 37 not taking any recordings (the latter were considered withdrawals) (Figure S4; Tables S4 and S5). Of the 1923, per protocol, population mean age was 70.2 (SD, 9.4), 958 (49.8%) were women, and mean CHA_2_DS_2_-VASc was 3.2 (SD, 1.0). In accordance with the prespecified recruitment plan, 1021 (53.1%) had high FIND-AF 2.0 risk (Table 3; Figure S5).

**Table 3.**
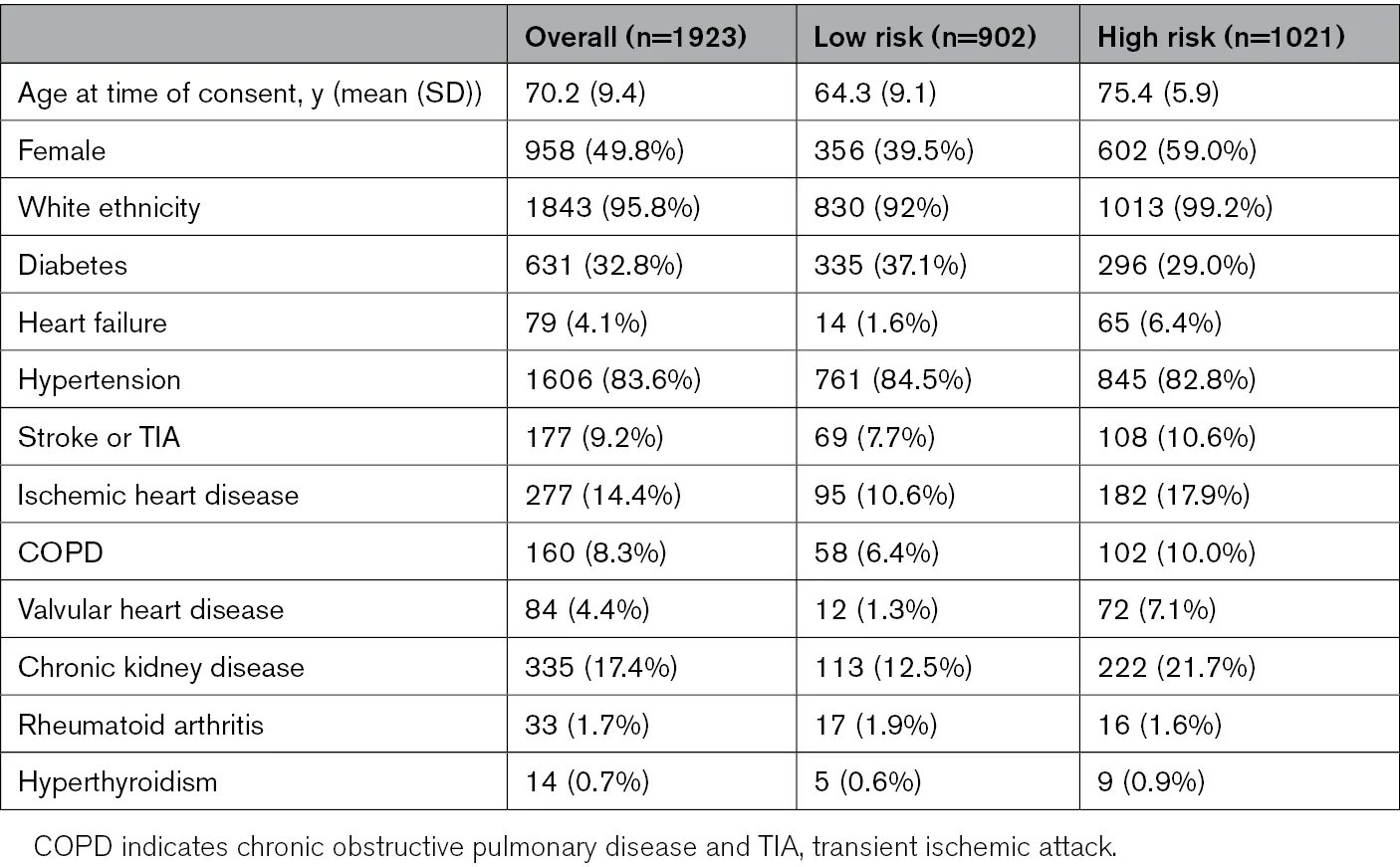
Characteristics of the Prospective Study Population at Baseline

The final intermittent ECG monitoring record was May 19, 2025. One thousand eight hundred eighty-four (98.0%) participants took at least 25 recordings, and 1732 (90.1%) took at least 50 recordings, with a mean of 74.8 (SD, 19.4) recordings per participant. The distribution of the number of recordings was similar between high and low FIND-AF 2.0 risk (Figure S6).

#### Primary Outcome

**Figure 2.**
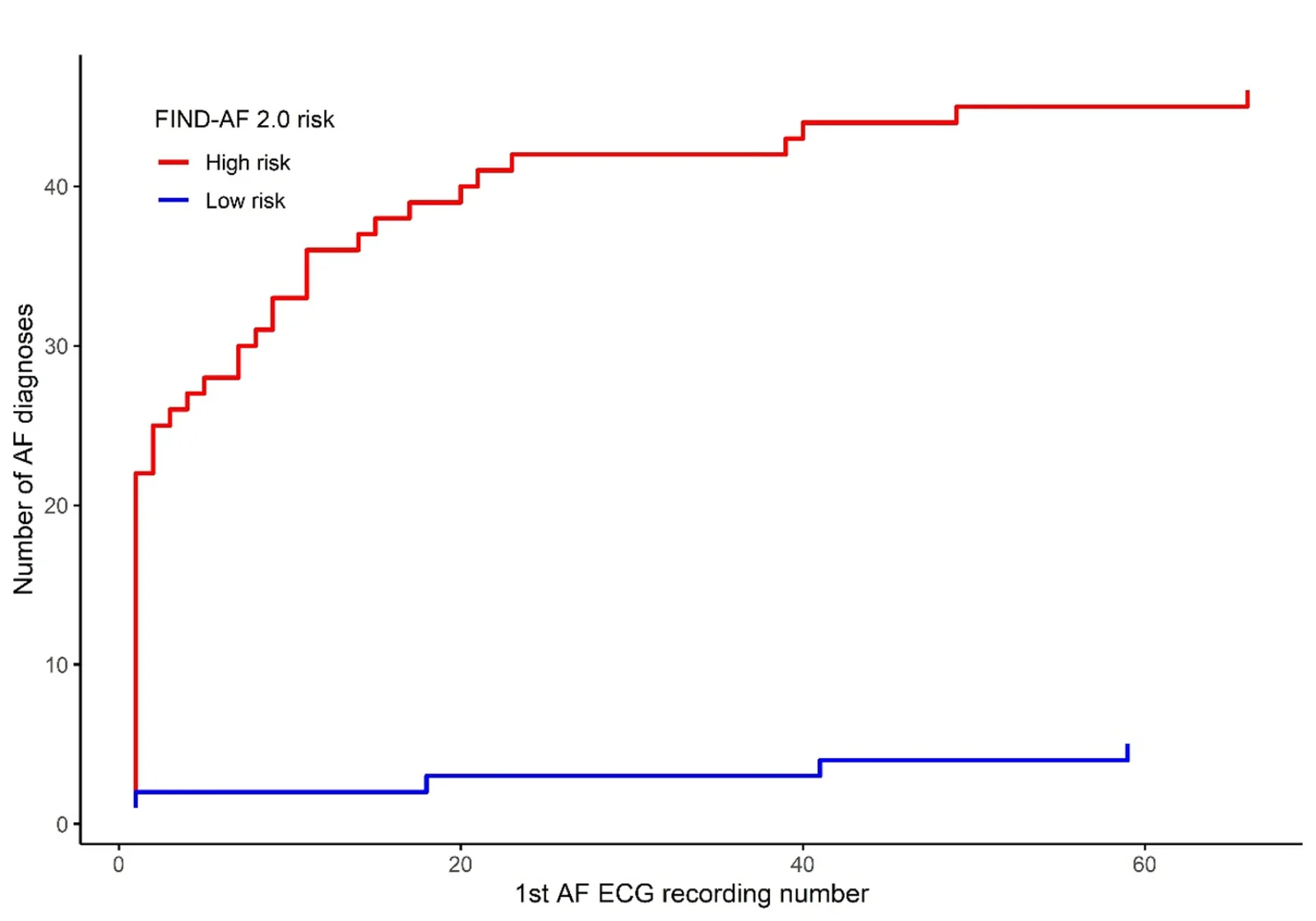
Cumulative incidence curve of atrial fibrillation (AF) diagnosis during a period of intermittent ECG monitoring, stratified by Future Innovations in Novel Detection of Atrial Fibrillation (FIND-AF) 2.0 risk.

During screening, 51 participants met the primary outcome, including 5 of 902 (0.6%) with low FIND-AF 2.0 risk and 46 of 1021 (4.5%) with high FIND-AF 2.0 risk (OR, 8.46 [95% CI, 3.35–21.40]; *P*<0.001). The timing of AF diagnosis in relation to the number of ECG recordings is shown in Figure 2. On the first recorded ECG, AF was diagnosed in 22 of 1021 participants with high FIND-AF 2.0 risk (2.1%) and 2 of 902 participants with low FIND-AF 2.0 risk (0.2%, *P<*0.01). Among participants who had AF detected on their first ECG, the median AF burden was 86.3% (interquartile range, 39.8%–98.2%).

The proportion of recordings with AF among those diagnosed with AF demonstrated a bimodal distribution between high and low AF burden (Figure S7). The median AF burden among AF cases detected with high FIND-AF 2.0 risk was 33.4% (interquartile range, 5.1%–91.6%) compared with 6.5% (3.6%–10.9%) in detected cases with low FIND-AF 2.0 risk (*P=*0.18) (Figure S8), with 14 of 46 (30.4%) detected AF cases with high FIND-AF 2.0 risk having AF in >80% of ECG recordings.

At the prespecified FIND-AF 2.0 threshold (≥0.0053), sensitivity was 90.2% (95% CI, 79.0%–95.7%), PPV 4.5% (95% CI, 3.4%–6.0%), and NPV 99.4% (95% CI, 98.7%–99.8%). AF diagnostic yield was plotted across the FIND-AF 2.0 scores, and though PPV was higher at FIND-AF 2.0 thresholds of 0.0060 and 0.0070 (4.7% and 4.7%, respectively) the sensitivity was lower (80.4% and 64.7%, respectively) (Table S6; Figure S9).

While CHA_2_DS_2_-VASc ≥2 for men and ≥3 for women identified all AF cases, the PPV was lower at 2.7% (Table S7). The highest PPV with CHA_2_DS_2_-VASc was 3.8% at a threshold of ≥3 for men and ≥4 for women (the threshold used in the AMALFI (Active Monitoring for Atrial Fibrillation) and EQUAL-AF RCTs)^22,23^ but with a lower sensitivity (72.5%). For C_2_HEST, a threshold of ≥1 had high sensitivity (96.1%) but a lower PPV of 2.7%, while C_2_HEST ≥3 would provide a PPV of the same magnitude as FIND-AF 2.0 (4.5%) but with low sensitivity (54.9%) and missing 23 of the 51 AF cases detected during intermittent ECG monitoring (Table S8). CHARGE-AF could be calculated for 49.7% of participants who had complete data. Participants with complete CHARGE-AF data had a lower detection rate of AF than those without complete CHARGE-AF data (1.3% versus 4.0%, *P*<0.001). At a CHARGE-AF threshold of 0.11, the PPV was 1.9%, NPV 99.7%, and sensitivity 91.7% (Table S9). Using an age of 75 and 76 years to guide screening (the criteria used in the STROKESTOP RCT)^19^ achieved a PPV of 3.0% but had very low sensitivity (13.7%) (Table S10). When adjusted for disease prevalence, NPV was higher for all approaches studied (Tables S6 through S10).

The F1 score was highest for FIND-AF 2.0 at the prespecified threshold (0.0858), followed by C_2_HEST ≥3 (0.0824), CHA_2_DS_2_-VASc ≥3 for men and ≥4 for women (0.0723) 75 and 76 years old (0.0470), and CHARGE-AF (0.0377) (Table S11). Subgroup analysis suggested that AF detection was higher in older patients (≥75 years) and in men compared with women across different approaches to guide AF screening, but this should be interpreted with caution given the low number of AF diagnoses in each subgroup (Tables S6 through S9).

#### Secondary Outcome

Of 51 patients newly diagnosed with AF during intermittent ECG monitoring, 49 (96.1%) were treated with oral anticoagulants by their primary care physician.

#### Safety

There were two incidental findings: 2:1 atrioventricular block in 1 patient and broad complex tachycardia in another.

### Association of FIND-AF 2.0 Risk Score With Ischemic Stroke in Patients With AF Not Taking Anticoagulants

Among 229 565 patients diagnosed with AF in the FinACAF registry, the nonanticoagulant rate of ischemic stroke was 6.0 events per 100 patient-years in those with high FIND-AF 2.0 risk and 1.7 per 100 patient-years in those with low FIND-AF 2.0 risk (incidence rate ratio, 3.43 [95% CI, 3.14–3.74) as compared with 5.9 events per 100 patient-years in those with high CHA_2_DS_2_-VASc risk compared with 1.2 events per 100 patient-years in those with low or intermediate CHA_2_DS_2_-VASc risk (incidence rate ratio, 5.08 [95% CI, 4.55–5.67) (Figure S10).

## DISCUSSION

This study developed and validated a parsimonious multivariable machine learning prediction model, FIND-AF 2.0, in almost 13 million patients from routine care cohorts across 5 countries and 3 continents and prospectively tested it in almost 2000 patients. FIND-AF 2.0 could identify a subpopulation for increased AF detection during screening among individuals with elevated CHA_2_DS_2_-VASc score. Furthermore, a third of AF cases detected during screening in those with high FIND-AF 2.0 risk had a high AF burden, and a high FIND-AF 2.0 risk is associated with a high rate of ischemic stroke occurrence in patients with untreated AF. To our knowledge, this is the largest and most geographically diverse study to develop, externally validate, and prospectively validate a prediction model to guide AF screening.

AF screening trials have so far been guided predominantly by age or elevated CHA_2_DS_2_-VASc score.^3^ The use of CHA_2_DS_2_-VASc to guide AF screening is not unreasonable given that it incorporates variables available with high completeness in routine EHR data and simultaneously establishes eligibility for anticoagulation treatment if AF is detected. In the prospective study, FIND-AF 2.0 achieved a higher diagnostic yield, sensitivity, and F1 score for new AF during ECG monitoring compared with the age-based approach used for the STROKESTOP RCT and the CHA_2_DS_2_-VASc-based criteria used for the AMALFI and EQUAL-AF RCTs.^1,3,19,22,24^ Optimizing patient selection for AF screening could improve the yield of new AF diagnoses and reduce unnecessary health care costs and patient anxiety, which is potentially clinically impactful for a population-based intervention such as AF screening. While age, CHA_2_DS_2_-VASc, and C_2_HEST can be mentally calculated to inform decisions on ECG monitoring for AF, FIND-AF 2.0 needs to be applied through existing health data systems. Nonetheless, for nationwide AF screening, a central EHR system or insurance database in which FIND-AF 2.0 could be applied would be required to identify eligible patients.

Alternative approaches have been studied for optimizing patient selection for AF screening, though direct comparison of yields of new AF with each approach is challenging because of differences in the intervention applied to detect undiagnosed AF. A study within the Mayo Clinic network showed that higher AF risk identified by artificial intelligence ECG was associated with a higher diagnostic yield for AF during screening compared with a lower risk,^13^ but the usefulness of this approach outside of this specific hospital network is unknown. A secondary analysis of the VITAL-AF RCT showed that, although a high artificial intelligence ECG risk was associated with higher AF detection rates, artificial intelligence ECG could be applied to only 55% of the participants.^25^ NT-proBNP (N-terminal pro-B-type natriuretic peptide)–guided AF screening in older patients demonstrated a similar high yield of new AF in the STROKESTOP II RCT,^26^ with an ECG monitoring protocol similar to that used in this study. However, assessment of NT-proBNP requires point-of-care testing, adding expense and inconvenience.

Multiple multivariable risk prediction models have been developed or validated for incident AF in EHRs.^7,27^ In retrospective studies, the prediction accuracy for models such as EHR-AF and CHARGE-AF is impressive.^12,27^ These models, however, require variables such as height, weight, and blood pressure that are often missing in routine nationwide community-based EHR systems. In our United Kingdom primary care dataset, we found that only 22% and 34% of patients ≥30 years old without known AF had complete data for the CHARGE-AF and EHR-AF scores, respectively (Supplemental Methods), and a previous validation in routine Dutch primary care data demonstrated only 17% of patients ≥40 years old without known AF had complete data for calculation of CHARGE-AF.^21^ In our prospective study, fewer than half of the participants had complete data for calculation of CHARGE-AF, those with incomplete data had higher rates of AF detection on screening, and the F1 score for CHARGE-AF was lower than FIND-AF 2.0. By contrast, FIND-AF 2.0 is applicable to international EHRs because it uses a parsimonious set of variables robust to missingness in routine data. We developed an application programming interface to enable FIND-AF 2.0 to be used in international EHR and insurance databases (TA/PPA/25/215005) and achieved regulatory approval for the model to be used in clinical practice in the United Kingdom (NIHR204580), with current piloting of the use of FIND-AF in routine National Health Service practice.

Whether AF screening should be conducted in clinical practice is controversial. Although the European Society of Cardiology guidelines give a class IIa recommendation for population-based screening for AF using a noninvasive ECG-based approach in individuals ≥75 years or ≥65 years old with additional stroke risk factors,^5^ this is not shared by the US Preventative Services Task Force in the absence of compelling evidence that AF screening improves clinical outcomes.^28^ We chose to use the intermittent ECG screening protocol used in the STROKESTOP RCT^1,19^ even though intermittent monitoring likely underestimates true AF incidence^29,30^ because STROKESTOP is the only RCT to show a (modest) benefit in clinical outcomes from AF screening,^19^ possibly because short episodes of AF, which have little impact on clinical outcomes, are not identified.^31^ Detection of AF during FIND-AF 2.0–guided AF screening in our prospective study did translate to prescription of oral anticoagulants by primary care physicians. Furthermore, a third of detected cases with a high FIND-AF 2.0 risk had a high AF burden, and there may be opportunities for comorbidity management and rhythm control to prevent progression of AF,^32^ which has been associated with worse outcomes.^33,34^ However, it is unclear whether treating screen-detected AF improves clinical outcomes. The SAFER RCT randomizing 82 000 people ≥70 years old not on oral anticoagulants to AF screening or routine care for the primary outcome of stroke is due to report in 2027.^14^ Prospective randomized assessment is required to determine whether FIND-AF 2.0–guided screening and comprehensive AF care improve clinical outcomes.

### Limitations

First, there may be misclassification of predictor variables in routine EHR data, including under-recording of diagnoses. The presence of nonrecorded diseases that impact AF genesis may lead to patients being classified as low risk. Second, in the development and external validation of FIND-AF 2.0, the AF outcome was defined by coded diagnoses in routine EHR datasets. Thus, underestimation of AF incidence is possible, since there will have been individuals with unrecorded asymptomatic AF. Furthermore, the association of predictor variables to newly diagnosed AF may be over- or underestimated if asymptomatic AF cases are more likely to be diagnosed in hospital settings during treatment or procedures for other conditions. The choice of a 6-month prediction horizon also limited the number of new cases of AF in retrospective EHR data but was advised by the FIND-AF patient advisory group and scientific advisory board to most accurately reflect prediction that is relevant to AF screening. Nevertheless, FIND-AF 2.0 did show a good ability on prospective evaluation to differentiate high- and low-risk individuals for ECG monitoring for the purpose of AF detection. Third, variables that are associated with AF genesis were not included in the FIND-AF 2.0 model due to missingness in routine data. However, the prediction performance for the parsimonious FIND-AF 2.0 model was not statistically different compared with the previously developed FIND-AF model, which included a larger selection of variables.^8^ Fourth, the incorporation of predictor variables as binary variables (hypertension diagnosis present or absent) rather than incorporation of more granular or longitudinal information (such as changes in blood pressure recordings) may impact the predictive ability of FIND-AF 2.0. However, observations and laboratory measures are often missing in routinely collected EHRs, and so scalability was prioritized by limiting variables to those that can be captured from diagnostic code lists. Fifth, our prospective study did not randomize participants in different risk strata to screening or usual care, and so we cannot say for certain that FIND-AF 2.0–guided screening increases AF detection compared with routine care. However, other studies of systematic AF screening have demonstrated increased AF detection compared with routine care.^3^ Sixth, the low number of detected AF cases in the low-risk group brings uncertainty to the OR for AF detection between high and low risk. Furthermore, the low overall number of events (51) means that there is overlap of the confidence intervals of PPV, NPV, and sensitivity between approaches to guide AF screening and that there is a lack of power to assess for variation in performance across subgroups. The F1 score was highest for FIND-AF 2.0, and for a population-based intervention, such as AF screening, that may be applied across millions of participants, optimizing the balance of detection and not missing cases is of utmost importance. Determining that FIND-AF 2.0 increases detection of AF compared with the CHA_2_DS_2_-VASc and C_2_HEST scores beyond statistical doubt would require a larger RCT. Seventh, the optimum FIND-AF 2.0 threshold to guide AF screening may vary in different geographies and requires prospective assessment. Eighth, stroke risk after AF diagnosis may have decreased since the FinACAF registry period ended, and the exact magnitude of risk for ischemic stroke for screen-detected AF may be smaller than in this cohort with clinical AF.^35^ Ninth, in the future, it may be possible to implement more sophisticated modeling and variable selection at scale across nationwide EHRs. The approach taken here was chosen through consensus design with a patient advisory group and scientific advisory board to enhance implementation within current EHR structures and informed by a systematic review of the literature.^7^

### Conclusions

FIND-AF 2.0, a parsimonious machine learning multivariable prediction model, on retrospective evaluation across 13 million EHRs and prospective evaluation in almost 2000 patients, demonstrated the ability to identify a high-risk subpopulation to guide more efficient AF screening. Whether FIND-AF 2.0–guided screening improves clinical outcomes requires prospective evaluation.

## ARTICLE INFORMATION

### Acknowledgments

The authors thank Tom Slater for providing a blinded review of the ECG recordings and AF diagnoses in the prospective interventional validation study and Matthew Duke for providing advice on primary care electronic health record searches. The authors also thank all members of the Libin Precision Medicine Initiative for support in preparing data resources used for the Calgary patients and all patients who participate in the Cardiovascular Imaging Registry of Calgary for their contributions to cardiovascular research.

### Disclosures

Dr Gale reports honoraria from AstraZeneca, Amgen, Bayer, Boehringer-Ingelheim, Daiichi Sankyo, Menarini, and Novartis. He has received educational and research grants from BMS, Abbott Inc, the British Heart Foundation, National Institute of Health Research, Horizon 2020, and the European Society of Cardiology outside the submitted work. Dr Camm reports personal fees from Abbott, Bayer, Sanofi, Milestone, Boston Scientific, InCarda, and Menarini. Dr Nakao was previously employed by the Department of Digital Health and Epidemiology, an industry-academia collaboration course supported by Eisai, and Kyowa Kirin and reports a study grant from Shimadzu Co and Bayer outside of this work. Dr Kawakami has received research funds from AstraZeneca, Eisai Co Ltd, Kyowa Kirin Co Ltd, OMRON Corporation, and Toppan Inc; consulting fees from Advanced Medical Care Inc, JMDC Inc, Ubicom Holdings Inc, Santen Pharmaceutical Co Ltd, and Shin Nippon Biomedical Laboratories Ltd; executive compensation from Cancer Intelligence Care Systems Inc and Mytreya Inc; honoraria from Kyoto University Original Co Ltd, Pharma Business Academy Ltd, Shionogi & Co Ltd, Taisho Pharmaceutical Co Ltd, and Sansho Co Ltd. Dr Lip is a consultant and speaker for BMS/Pfizer, Boehringer Ingelheim, Daiichi-Sankyo, Anthos (no fees are received personally). He is co-PI of the AFFIRMO project on multimorbidity in AF (grant agreement 899871), the TARGET project on digital twins for personalized management of atrial fibrillation and stroke (grant agreement 101136244), and the ARISTOTELES project on artificial intelligence for management of chronic long-term conditions (grant agreement 101080189), which are all funded by the EU’s Horizon Europe Research & Innovation Program. Dr Mercer reports personal fees from Abbott. Dr Freedman reports honoraria from BMS/Pfizer and Omron. Dr Langén received speaker fees from Boehringer-Ingelheim. Dr Airaksinen received speaker fees from Bayer, Pfizer, and Boehringer-Ingelheim. Dr Lehto received consulting fees from BMS-Pfizer Alliance, Bayer, Boehringer-Ingelheim, AstraZeneca, Organon, and MSD; speaker fees from BMS-Pfizer Alliance, Bayer, Boehringer-Ingelheim, AstraZeneca, Organon, and MSD; and support to attend meetings from Bayer. Dr Flewitt reports shareholdings in Cohesic Inc. Dr White reports research funding from Siemens Healthineers, Bristol Meyers Squibb, Pfizer Inc, and Circle Cardiovascular Inc and shares in Cohesic Inc.

### Supplemental Material

Supplemental Methods

Tables S1–S11

Figures S1–S10

## Supplementary Material
