# Supplementary material for: Risk-Guided Screening for Atrial Fibrillation Using Electronic Health Records
Source: Circulation. 2026 Jul 28;154(8):707–17. doi: 10.1161/CIRCULATIONAHA.126.079391 (PMC13506190; doi:10.1161/CIRCULATIONAHA.126.079391)

SUPPLEMENTAL MATERIAL

Risk-guided screening for atrial fibrillation using electronic health records

Ramesh Nadarajah*, PhD; Prof Jianhua Wu*, PhD; Ali Wahab, MBBS; Catherine Reynolds, BSc; Mohammad Haris, MRes; Tobin Joseph, MBBS; Keerthenan Raveendra, MPhil; Ben Hurdus, MBBS; Khalid Kazi MBBS; Sheena Bennet, RN; Chris Hayward, PhD; Ben Mercer, PhD; Jing Kang, PhD; Chenyi Gao, PhD; Yoko M Nakao, MD; Professor Koji Kawakami, MD; Carlin Chang, MD; Professor Abraham Wai, MD; Professor Jiandong Zhou, PhD; Professor Gary Tse, PhD; Talish Razi Benita, MPH; Professor Lior Rokach, PhD; Ronen Arbel, PhD; Professor Moti Haim, MD; Professor Doron Zahger, MD; Dina Labib, PhD; Jacqueline Flewitt, MSc; Professor James A White, MD; Konsta Teppo, PhD; Mika Lehto, PhD; Ville Langén, PhD; Aleksi K Winstén, MSc; Professor KE Juhani Airaksinen, PhD; Jari Haukka, PhD; Olli Halminen, PhD; Jukka Putaala, PhD; Professor Juha Hartikainen, PhD; Professor Miika Linna, PhD; Professor Ben Freedman, PhD; Emma Svennberg, PhD; Professor A John Camm, MD; Professor Gregory Y H Lip, MD; Professor Chris P Gale PhD

Supplemental Methods 3

Data sources and study populations for retrospective prediction modelling 3

Outcomes 5

Code lists to identify outcomes in CPRD-GOLD 6

Definition of risk factors 7

Sample size 8

Statistical analyses 9

Prediction models developed for newly diagnosed atrial fibrillation in the community 11

Missingness of variables included in prediction models developed for newly diagnosed atrial fibrillation in the community 12

Original set of variables in FIND-AF 13

Association of FIND-AF 2.0 risk score with ischaemic stroke in patients with AF not anticoagulated 17

Supplemental Tables 19

Supplementary Table 1: Characteristics of patients in derivation and external validation cohorts stratified by outcome of AF during follow up 19

Supplementary Table 2: Prediction performance (AUROC) of FIND-AF 2.0, CHA_2DS_2-VASc and C_2HEST for newly diagnosed AF within six months across the included cohorts stratified by sex 20

Supplementary Table 3: Area under the precision-recall curve (PR-AUC) of FIND-AF 2.0, CHA_2DS_2-VASc and C_2HEST for newly diagnosed AF within six months 21

Supplementary Table 4: Comparison between prospective interventional non-randomised validation study participants and withdrawals 22

Supplementary Table 5: Participating general practice sites in the prospective interventional non-randomised validation study 23

Supplementary Table 6: Sensitivity, negative and positive predictive values for newly diagnosed atrial fibrillation at different FIND-AF 2.0 score thresholds in the prospective interventional non-randomised validation study 24

Supplementary Table 7: Sensitivity, negative and positive predictive values for newly diagnosed atrial fibrillation at different CHA_2DS_2-VASc thresholds in the prospective interventional interventional non-randomised validation study 26

Supplementary Table 8: Sensitivity, negative and positive predictive values for newly diagnosed atrial fibrillation diagnosis at different C_2HEST thresholds in the prospective interventional non-randomised validation study 28

Supplementary Table 9: Sensitivity, negative and positive predictive values for newly diagnosed atrial fibrillation at different CHARGE-AF score thresholds in the prospective interventional non-randomised validation study 30

Supplementary Table 10: Sensitivity, negative and positive predictive values for newly diagnosed atrial fibrillation diagnosis using age in the prospective interventional non-randomised validation study 33

Supplementary Table 11: Confusion matrix summary for the different enrichment approaches in the prospective study. 34

Supplemental Figures 35

Supplementary Figure 1: Flow chart of process for selecting variables for the FIND-AF prediction model 35

Supplementary Figure 2: Variable importance in the FIND-AF prediction model quantified by mean decrease in Gini impurity 37

Supplementary Figure 3: Receiver operating characteristic curve for the prediction of newly diagnosed atrial fibrillation diagnosis within the next six months for FIND-AF, CHA_2DS_2-VASc, and C_2HEST across included cohorts 38

Supplementary Figure 4. Prospective interventional non-randomised validation study flowchart 39

Supplementary Figure 5: Distribution of FIND-AF 2.0 score across prospective interventional non-randomised validation study participants stratified by high and low risk 40

Supplementary Figure 6: Distribution of number of ECG recordings taken per participant stratified by high and low risk 41

Supplementary Figure 7: Percentage of recordings with AF amongst detected AF cases 42

Supplementary Figure 8. Proportion of recordings with AF for patients with newly diagnosed AF stratified by FIND-AF 2.0 risk. 43

Supplementary Figure 9: Estimated AF diagnostic yield from intermittent ECG monitoring across FIND-AF 2.0 risk scores 44

Supplementary Figure 10: Cumulative incidence of ischaemic stroke within 12 months for patients with atrial fibrillation not anticoagulated in the FinACAF study stratified by FIND-AF2.0 risk and CHA_2DS_2-VASc 45

Supplemental Methods

Data sources and study populations for retrospective prediction modelling

Clinical Practice Research Datalink-GOLD

Clinical Practice Research Datalink (CPRD)-GOLD contains information from general practices in the United Kingdom using Vision electronic health record software, from Jan 1, 1985, to Nov 30, 2018. The CPRD-GOLD database contains anonymised patient data from approximately 7% of the UK population and is broadly representative in terms of age, sex, and ethnicity. These data were linked to secondary care admission records from Hospital Episodes Statistics Admitted Patient Care (HES-APC) data and Office for National Statistics (ONS) mortality data. Scientific approval for this study was given by the CPRD-GOLD Independent Scientific Advisory Committee (ISAC).

Participants were men and women aged 30 years and older, contributing to data between Jan 1, 1998, and November, 2018. Patients were eligible for inclusion if their record was labelled as acceptable by CPRD-GOLD quality control, approved for CPRD-GOLD and Hospital Episodes Statistics linkage, if they were registered with their general practice for at least 12 months, and if they had no prior history of atrial fibrillation (AF) or atrial flutter (AFl). Participants entered the cohort at the point of registration without a concurrent diagnosis of AF. Individuals were censored to a diagnosis of AF or AFl, withdrawal from CPRD-GOLD, or six months, whichever came first.

Japan Medical Insurance Claims Database

Data were extracted from the Japan Medical Insurance Claims Database (JMDC), a combined database of health insurance claims from multiple insurance providers in Japan. JMDC records all diagnostic, outpatient, inpatient treatment and medications for individual with health insurance. Individuals are aged between 30-74 and entered the database from 2005 through to October 2023. The database includes monthly claims and uses International Classification of Disease, 10th edition (ICD-10) codes to record diagnoses, procedural and prescription codes. JMDC data has been used in numerous clinical studies previously, and has also been shown to be comparable to national database data. Patients without a known or concurrent diagnosis of AF or AFl were included.

Clinical Data Analysis and Reporting System

Data were extracted from the Clinical Data Analysis and Reporting System (CDARS), a population-based, administrative electronic medical records system in Hong Kong, China. CDARS records all diagnostic, procedural and prescription records of patients attending public healthcare institutions in Hong Kong, which serve an estimated 90% of the population. Diagnoses were encoded by International Classifications of Disease*, Ninth Revision* (ICD-9) codes regardless of the time of data entry, as ICD-10

has not been implemented in CDARS to date. CDARS has been used extensively in prior studies and shown to have good coding accuracy and data completeness. Entry to the cohort was between January 1 2000 and December 31 2003, with follow up till December 31 2019. Patients without a known or concurrent diagnosis of AF or AFl were included. Ethical approval for the study was provided by Institutional Review Board No. UW 23-339

Clalit Health Services (CHS)

CHS is a large health-care organisation which covers approximately two-thirds of the Israeli population. The CHS data repositories were previously described in COVID-19 studies. CHS covers approximately 52% of the Israeli population and is the largest of four health care organizations in Israel that provide mandatory health care. Participants with missing data regarding date of birth or sex were excluded from the study. We analysed patient-level data that were extracted from CHS electronic medical records. The CHS operational database includes sociodemographic data and comprehensive clinical information, such as coexisting chronic conditions, community-care visits, hospitalizations, medications, and results of laboratory tests and imaging studies. Diagnoses were identified using ICD-10 codes. Participants were men and women aged ≥30 years without a recorded diagnosis of AF or AFl.

Cardiovascular Imaging Registry of Calgary (CIROC)

CIROC is a clinical outcomes registry of the Libin Cardiovascular Institute, launched in January 2014, and routinely engages patients referred to cardiac imaging services and/or electrocardiograms in Southern Alberta, Canada. Patients provide informed consent for data usage. As of August 1st, 2024, around four million ECGs have been ethically sourced and matched to electronic health information from institutional electronic health information repositories to obtain relevant demographics, laboratory, pharmacy, 12-lead ECG, Holter, and ICD-10 coded diagnostic and procedural data. To be included in this study the individuals were required to have no diagnostic code in their records for AF or AFl and an ECG or holter monitor with sinus rhythm.

Outcomes

The primary outcome was newly diagnosed AF or AFl, collectively referred to as AF.

CPRD-GOLD

Diagnoses in primary care were identified using Read codes in CPRD-GOLD and in secondary care and on death certificates with the tenth revision of the ICD-10 codes in HES-APC and ONS mortality data. We defined newly diagnosed AF diagnosis as the first record of AF in primary care or hospital admission records from any diagnostic position. Diagnostic coding for AF in CPRD-GOLD has been shown to be consistent and valid, with a positive predictive value of 98%.

JMDC

Diagnoses of AF were based on ICD-10 codes. We included cases from any diagnostic position.

CDARS

Diagnoses of AF were based (ICD-9) codes. We included cases from any diagnostic position.

CHS

Diagnoses of AF were based on ICD-10 codes. We included cases from any diagnostic position.

CIROC

Diagnoses of AF were based on ICD-10 codes. We included cases from any diagnostic position.

Code lists to identify outcomes in CPRD-GOLD

Code	Description
Readcodes	
G573200	Paroxysmal atrial fibrillation
G573400	Permanent atrial fibrillation
G573500	Persistent atrial fibrillation
3272	ECG: atrial fibrillation
G573000	Atrial fibrillation
G573300	Non-rheumatic atrial fibrillation
G573.00	Atrial fibrillation and flutter
G573z00	Atrial fibrillation and flutter NOS
3273	ECG: atrial flutter
G573100	Atrial flutter
ICD-10 codes	
I48	Atrial fibrillation and flutter

Definition of risk factors

CPRD-GOLD

In CPRD-GOLD covariates were defined by any occurrence of relevant Read code at any time point before the index date. Diagnosis code lists from the CALIBER code repository were used for each condition. For comorbidities that were candidate predictors, if medical codes were absent in a patient record we assumed that the patient did not have that diagnosis. Age and sex were recorded for every individual in the dataset.

JMDC

Identified with ICD-10 codes.

CDARS

Identified with ICD-9 codes.

CHS

Identified with ICD-10 codes.

CIROC

Identified with ICD-10 codes.

Sample size

For 20 parameters to be included in the prediction model and the Cox-Snell generalised R^2 is assumed to be 0.05. A total of 3,699 patients will be required to meet Riley's criterion (1) with global shrinkage factor of 0.95; this sample size also ensures a small absolute difference ($\Delta<0.05$) in the apparent and adjusted Nagelkerke R2 (Riley's criterion (2)) and ensures precise estimate of overall risk with a margin of error <0.001 (Riley's criterion (3)). In the derivation cohort there were 2,081,139 participants with 7,386 new AF cases and in the validation cohorts there were 10,739,103 participants with 21,110 new AF cases. Therefore, the number of patients provided sufficient statistical power to develop and validate a multivariable prediction model with the predefined precision and accuracy.

Statistical analyses

We calculated descriptive statistics for baseline characteristics in the cohorts. Continuous variables were reported as mean ± standard deviation (SD). Categorical variables were reported as frequencies with corresponding percentages.

In the model development study we explored a random forest machine learning approach based on our previous study, with the development dataset randomly split into a development (80%) hold out validation (20%) cohort%) using the Mersenne Twister pseudorandom number generator..

Random forest is a machine learning method consisting of many individual decision trees that operate as an ensemble. The model produces an output of AF risk quantified between 0 and 1. To address class imbalance because of low AF incidence within short time window, we applied class weighting during random forest model training. We assigned weights inversely proportional to class frequencies, ensuring greater penalisation for misclassification of minority class events. This method improves sensitivity to rare outcomes while maintaining overall model integrity without resampling.

The Random forest model was trained using the following parameters. Each decision tree used Gini impurity, commonly used in classification and regression tree (CART) algorithms, to measure the split quality. The minimum impurity split threshold for each node, above which a node will split into two or more branches, was set to 10^{-7}. The minimum number of samples required to split a node was set to two. The minimum samples per leaf was set to one. All the algorithm's hyperparameters were tuned using the grid search method, in which all possible combinations were evaluated, resulting in 1000 trees, mtry = 8 (the number of random features to consider in each tree) and nodesize = 12 (number of patients classified at that node). Variable importance was quantified using the Gini impurity measure, which is a measure of how each variable contributes to the homogeneity of nodes and leaves in the resulting random forest.

Model performance was determined using the validation dataset with internal bootstrap validation with 200 samples. The area under the received operating characteristic curve (AUROCC) was used to evaluate predictive ability (concordance index) with 95% confidence intervals (CIs) calculated using the DeLong method. AUROC values of <0.60, 0.60-0.70, 0.70-0.80, and >0.80 were defined a priori as inadequate, adequate, good and excellent based on prior publications. Given the rarity of the outcome, area under the precision-recall curve (PR AUC) was additionally reported as a complementary measure within each dataset. Calibration was assessed by the calibration slope. We calculated the Brier score, a measure of both discrimination and calibration, by taking the mean squared difference between predicted probabilities and the observed outcome. We conducted subgroup analysis in men and women. To compare the AUROCs between men and women, we used the DeLong test, a nonparametric approach that evaluates the difference between two correlated or independent ROC curves.

Researchers at JMDC (YMN), CHS (RA, LR, TRB), CIROC (JAW, DL) and CDARS (GT, JZ) conducted external validation using the same methodology.

Prediction models developed for newly diagnosed atrial fibrillation in the community

Model	Variables
CHARGE-AF	Age, race, height, weight, SBP, DBP, current smoking, use of antihypertensive medication, diabetes, myocardial infarction, heart failure
ARIC	Age, race, height, smoking status, DBP, use of antihypertensive medication, praecordial murmur, left atrial enlargement, left ventricular hypertrophy, diabetes, coronary heart disease, heart failure
Framingham-AF	Age, sex, BMI, SBP, use of antihypertensive medication, PR interval, significant murmur, heart failure
PULsE-AI	Age, sex, race, smoking status, hypertension, use of antihypertensive medication, heart failure, ischaemic heart disease, congenital heart disease, myocardial infarction, left ventricular hypertrophy, Type 1 diabetes mellitus, Type 2 diabetes mellitus<br>Height, weight, BMI, SBP, DBP, within last 12 months
$HARMS_2$-AF	Age, hypertension, BMI, sex, smoking status, alcohol consumption, sleep apnoea
MHS	Age, sex, BMI, SBP, use of antihypertensive medication, chronic lung disease, myocardial infarction, peripheral arterial disease, heart failure, inflammatory disease
EHR-AF	Age, sex, smoking status, height, weight, DBP, hypertension, hyperlipidaemia heart failure, coronary heart disease, valvular disease, stroke, peripheral arterial disease, chronic kidney disease, hypothyroidism
Seirei	Age, waist circumference, DBP, alcohol consumption, heart rate, cardiac murmur
Suita	Age, hypertension, SBP, BMI, alcohol consumption, non-HDL-C, cardiac murmur, smoking status, arrhythmia
WHS	Age, weight, height, systolic blood pressure, alcohol consumption, smoking status

Models collected from two previous systematic reviews of prediction models for newly diagnosed AF in the community and community-based electronic health records, and European Society of Cardiology guidelines

ARIC, Atherosclerosis Risk In Communities; BMI, body mass index; CHARGE-AF, Cohorts for Heart and Aging Research in Genomic Epidemiology-Atrial Fibrillation; DBP, diastolic blood pressure; EHR-AF, Electronic Health Record-Atrial Fibrillation; $HARMS_2$-AF, Hypertension, Age, Raised BMI, Male sex, Sleep apnoea, Smoking, Alcohol; HDL-C, high density lipoprotein cholesterol; MHS, Maccabi Healthcare Services; PULsE-AI, Prediction of Undiagnosed atriaL fibrillation using a machinE learning AlgorIthm; SBP, systolic blood pressure; WHS, Women’s Health Study

Missingness of variables included in prediction models developed for newly diagnosed atrial fibrillation in the community

**Model**	**Proportion of patients with at least one record in each variable in routine EHR data**															**Proportion of patients with full dataset to calculate risk score**
	Height	Weight	BMI	Waist circumference	SBP	DBP	Heart rate	Race	Smoking status	Alcohol consumption	Murmur	Left atrial enlargement	Left ventricular hypertrophy	PR interval	non-HDL-C	
CHARGE-AF	43.2%	56.4%	-	-	63.6%	63.6%	-	91.6%	75.1%	-	-	-	-	-	-	21.5%
ARIC	43.2%	-	-	-	63.6%	-	-	91.6%	75.1%	-	NA	NA	100.0%	-	-	0.0%
Framingham-AF	-	-	40.1%	-	63.6%	-	-	-	-	-	NA	-	-	NA	-	0.0%
PULsE-AI*	17.5%	36.1%	32.3%	-	47.8%	47.8%	-	91.6%	75.1%	-	-	-	-	-	-	10.9%
$HARMS_2$-AF	-	-	40.1%	-	-	-	-	-	75.1%	48.1%	-	-	-	-	-	31.5%
MHS	-	-	40.1%	-	63.6%	-	-	-	-	48.1%	-	-	-	-	-	28.2%
EHR-AF	43.2%	56.4%	-	-	-	63.6%	-	-	75.1%	-	-	-	-	-	-	33.6%
Seirei	-	-	-	43.7%	-	63.6%	29.7%	-	-	48.1%	NA	-	-	-	-	0.0%
Suita	-	-	40.1%	-	63.6%	-	-	-	75.1%	48.1%	NA	-	-	-	16.6%	0.0%
WHS	43.2%	56.4%	-	-	63.6%	-	-	-	75.1%	48.1%	-	-	-	-	-	19.4%

*Recorded within last 12 months

NA, variable is used in the risk assessment but not available in CPRD-GOLD.

ARIC, Atherosclerosis Risk In Communities; BMI, body mass index; CHARGE-AF, Cohorts for Heart and Aging Research in Genomic Epidemiology-Atrial Fibrillation; DBP, diastolic blood pressure; EHR-AF, Electronic Health Record-Atrial Fibrillation; HARMS2-AF, Hypertension, Age, Raised BMI, Male sex, Sleep apnoea, Smoking, Alcohol; HDL-C, high density lipoprotein cholesterol; MHS, Maccabi Healthcare Services; PULsE-AI, Prediction of Undiagnosed atriaL fibrillation using a machinE learning AlgorIthm; SBP, systolic blood pressure; WHS, Women's Health Study

Original set of variables in FIND-AF

Comorbidity associated with / predictive of atrial fibrillation	Categorisation	References and Rationale for categorisation
Demographics		
Age	-	Hindricks G, Potpara T, Dagres N, et al. 2020 ESC Guidelines for the diagnosis and management of atrial fibrillation developed in collaboration with the European Association of Cardio-Thoracic Surgery (EACTS). Eur Heart J 2020<br><br>Incidence of AF increases with age (therefore included as a continuous variable)
Sex	Men	Hindricks G, Potpara T, Dagres N, et al. 2020 ESC Guidelines for the diagnosis and management of atrial fibrillation developed in collaboration with the European Association of Cardio-Thoracic Surgery (EACTS). Eur Heart J 2020<br><br>AF is more common in men
	Women	
Ethnicity	Asian	Shen AY-J, Contreras R, Sobnosky S, et al. Racial/ethnic differences in the prevalence of atrial fibrillation among older adults—a cross-sectional study. J Natl Med Assoc 2010;102(10):906-14.<br><br>Chiang C-E, Zhang S, Tse HF, et al. Atrial fibrillation management in Asia: from the Asian expert forum on atrial fibrillation. Int J Cardiol 2013;164(1):21-32.<br><br>White, Asian, pacific Asian, and black ethnicities have different odds ratios of development of AF
	Black	
	Mixed	
	Other	
	Pacific Asian	
	White	
Alcohol use	Ex-	Samokhvalov AV, Irving HM, Rehm J. Alcohol consumption as a risk factor for atrial fibrillation: a systematic review and meta-analysis. European Journal of Preventive Cardiology 2010;17(6):706-12.<br><br>There is a monotonic dose-response relationship between alcohol consumption and AF incidence
	Light,	
	Moderate	
	Excess	
	Unspecified	
Smoking	Current	Heeringa J, Kors JA, Hofman A, et al. Cigarette smoking and risk of atrial fibrillation: the Rotterdam Study. Am Heart J 2008;156(6):1163-69.<br><br>Watanabe I. Smoking and risk of atrial fibrillation: Elsevier, 2018.<br><br>Current and ex-smokers are at increased risk of AF, with a higher risk in current smokers.
	Ex	
Weight	Obese	Lavie CJ, Pandey A, Lau DH, et al. Obesity and atrial fibrillation prevalence, pathogenesis, and prognosis: effects of weight loss and exercise. J Am Coll Cardiol 2017;70(16):2022-35.<br><br>Frost L, Hune LJ, Vestergaard P. Overweight and obesity as risk factors for atrial fibrillation or flutter: the Danish Diet, Cancer, and Health Study. The American journal of medicine 2005;118(5):489-95.<br><br>Lee S-R, Choi E-K, Park CS, et al. Direct oral anticoagulants in patients with nonvalvular atrial fibrillation and low body weight. J Am Coll Cardiol 2019;73(8):919-31.<br><br>Obesity is associated with a greater risk of AF than being overweight. Low body weight is associated with a higher risk of AF than normal weight.
	Overweight	
	Underweight	
Comorbidities		
Adult congenital heart disease	-	-

Anaemia	-	-
Cancer	Leukaemia	Thompson PA, Lévy V, Tam CS, et al. Atrial fibrillation in CLL patients treated with ibrutinib. An international retrospective study. Br J Haematol 2016;175(3):462-66.<br><br>Sorigue M, Gual-Capllonch F, Garcia O, et al. Incidence, predictive factors, management, and survival impact of atrial fibrillation in non-Hodgkin lymphoma. Ann Hematol 2018;97(9):1633-40.<br><br>Han H, Chen L, Lin Z, et al. Prevalence, trends, and outcomes of atrial fibrillation in hospitalized patients with metastatic cancer: findings from a national sample. Cancer medicine 2021;10(16):5661-70.<br><br>AF risk is higher in patients with leukaemia and lymphoma, especially treated with ibrutinib. Solid organ cancers (such as lung and colorectal cancer) are more likely to undergo surgery. Metastatic disease is associated with higher risk of AF compared to non-metastatic disease. Skin cancers other than melanoma have a lower risk of metastasis and hence AF.
	Lymphoma	
	Metastasis	
	Skin cancers other than melanoma	
	Solid organ	
Cardiac surgery	Valvular,	Greenberg JW, Lancaster TS, Schuessler RB, et al. Postoperative atrial fibrillation following cardiac surgery: a persistent complication. Eur J Cardiothorac Surg 2017;52(4):665-72.<br><br>Within overall cardiac surgical procedures incidence of post-operative AF is 35%, with variation by surgical procedure.
	Non-valvular	
Chronic kidney disease	Stage 1-2	Alonso A, Lopez FL, Matsushita K, et al. Chronic kidney disease is associated with the incidence of atrial fibrillation: the Atherosclerosis Risk in Communities (ARIC) study. Circulation 2011;123(25):2946-53.<br><br>Risk of AF increases as CKD stage worsens and if there is proteinuria
	Stage 3	
	Stage 4	
	Stage 5	
	Unspecified	
	Other	
COPD	-	-
Cerebrovascular accident	Intracerebral haemorrhage	Hindricks G, Potpara T, Dagres N, et al. 2020 ESC Guidelines for the diagnosis and management of atrial fibrillation developed in collaboration with the European Association of Cardio-Thoracic Surgery (EACTS). Eur Heart J 2020<br><br>Association with AF is higher for ischaemic strokes than haemorrhagic strokes
	Subarachnoid haemorrhage	
	Unspecified	
Diabetes Mellitus	Good control	Dublin S, Glazer NL, Smith NL, et al. Diabetes mellitus, glycemic control, and risk of atrial fibrillation. J Gen Intern Med 2010;25(8):853-58.<br><br>Poorer glycaemic control is associated with a higher risk of AF compared to better glycaemic control or no diabetes
	Poor control	
	Unspecified / secondary	
Deep venous thrombosis	-	-
Dyslipidaemia	-	-
Infective Endocarditis	-	-
Electrophysiology procedure affecting the atria	-	-
Gout	-	-
Hypertrophic cardiomyopathy	-	-
Heart failure	-	-
Hypertension	Poor control	Dzeshka MS, Shantsila A, Shantsila E, et al. Atrial fibrillation and hypertension. Hypertension 2017;70(5):854-61.<br><br>Poorer control of hypertension and end organ damage is associated with a higher risk of developing AF
	Unspecified / secondary	
Hyperthyroidism	-	-

Inflammatory bowel disease	-	-
Intensive care unit admission	-	-
Ischaemic heart disease	Chronic	Huxley RR, Lopez FL, Folsom AR, et al. Absolute and attributable risks of atrial fibrillation in relation to optimal and borderline risk factors: the Atherosclerosis Risk in Communities (ARIC) study. Circulation 2011;123(14):1501-08.<br><br>Pizzetti F, Turazza F, Franzosi M, et al. Incidence and prognostic significance of atrial fibrillation in acute myocardial infarction: the GISSI-3 data. Heart 2001;86(5):527-32.<br><br>There is a high risk of AF in the acute setting of myocardial infarction as well as evidence in the context of underlying chronic coronary syndromes.
	Myocardial infarction	
	Percutaneous coronary intervention	
Infection	Gastrointestinal	Gundlund A, Olesen JB, Butt JH, et al. One-year outcomes in atrial fibrillation presenting during infections: a nationwide registry-based study. Eur Heart J 2020;41(10):1112-19.<br><br>Chang T-Y, Chao T-F, Liu C-J, et al. The association between influenza infection, vaccination, and atrial fibrillation: A nationwide case-control study. Heart Rhythm 2016;13(6):1189-94.<br><br>Klein Klouwenberg PM, Frencken JF, Kuipers S, et al. Incidence, predictors, and outcomes of new-onset atrial fibrillation in critically ill patients with sepsis. A cohort study. Am J Respir Crit Care Med 2017;195(2):205-11.<br><br>In a cohort study among infections precipitating AF the order of risk is as follows: Pneumonia > sepsis > urinary tract infection > gastrointestinal infection
	Influenza	
	Respiratory	
	Sepsis	
	Urinary	
Left ventricular hypertrophy	-	-
Myocarditis	-	-
Obstructive sleep apnoea	-	-
Pulmonary embolus	-	-
Pericarditis	-	-
Pulmonary hypertension	-	-
Peripheral vascular disease	-	-
Rheumatological condition	Autoimmune connective tissue diseases	Lee E, Choi E-K, Jung J-H, et al. Increased risk of atrial fibrillation in patients with Behçet's disease: a nationwide population-based study. Int J Cardiol 2019;292:106-11.<br><br>Moon I, Choi E-K, Jung J-H, et al. Ankylosing spondylitis: a novel risk factor for atrial fibrillation—a nationwide population-based study. Int J Cardiol 2019;275:77-82.<br><br>Melduni RM, Cooper LT, Gersh BJ, et al. Association of Autoimmune Vasculitis and Incident Atrial Fibrillation: A Population-Based Case-Control Study. Journal of the American Heart Association 2020;9(18):e015977.<br><br>Naaraayan A, Meredith A, Nimkar A, et al. Arrhythmia prevalence among patients with Polymyositis-Dermatomyositis in the United States: an observational study. Heart Rhythm 2021<br><br>Songnan W, Shengma C. GW24-e2483 Catheter ablation of atrial fibrillation in patients with autoimmune rheumatic diseases. Heart 2013;99(Suppl 3):A197-A97.
	Rheumatoid arthritis	
	Spondyloarthropathies	
	Vasculitides	

		Giallafos I, Triposkiadis F, Oikonomou E, et al. Incident atrial fibrillation in systemic sclerosis: the predictive role of B-type natriuretic peptide. Hellenic J Cardiol 2014;55:313-21.<br><br>Pugnet G, Gouya H, Puéchal X, et al. Cardiac involvement in granulomatosis with polyangiitis: a magnetic resonance imaging study of 31 consecutive patients. Rheumatology 2017;56(6):947-56.<br><br>Lindhardsen J, Ahlehoff O, Gislason GH, et al. Risk of atrial fibrillation and stroke in rheumatoid arthritis: Danish nationwide cohort study. BMJ 2012;344<br><br>Each of the subtypes of rheumatological disease are associated with differing risks of development of AF. Here they have been categorised in clinical sub-type.
Surgery (non-cardiac)	Colorectal	Siu CW, Tung HM, Chu KW, et al. Prevalence and predictors of new-onset atrial fibrillation after elective surgery for colorectal cancer. Pacing Clin Electrophysiol 2005;28:S120-S23.<br><br>Onaitis M, D'Amico T, Zhao Y, et al. Risk factors for atrial fibrillation after lung cancer surgery: analysis of the Society of Thoracic Surgeons general thoracic surgery database. The Annals of thoracic surgery 2010;90(2):368-74.<br><br>Philip I, Berroëta C, Leblanc I. Perioperative challenges of atrial fibrillation. Current Opinion in Anesthesiology 2014;27(3):344-52.<br><br>Thoracic surgery is associated with the greatest risk of post-operative AF amongst non-cardiac surgeries followed by colorectal then vascular surgery
	Thoracic	
	Vascular	
Systemic Embolism	-	-
Valvular heart disease	Mitral stenosis / rheumatic valvular disease	Iung B, Leenhardt A, Extramiana F. Management of atrial fibrillation in patients with rheumatic mitral stenosis. Heart 2018;104(13):1062-68.<br><br>Levy S. Factors predisposing to the development of atrial fibrillation. Pacing Clin Electrophysiol 1997;20(10):2670-74.<br><br>Grigioni F, Avierinos J-F, Ling LH, et al. Atrial fibrillation complicating the course of degenerative mitral regurgitation: determinants and long-term outcome. J Am Coll Cardiol 2002;40(1):84-92.<br><br>The association of mitral stenosis and rheumatic valve disease with AF is greater than mitral regurgitation followed by diseases of other valves
	Non-mitral valve / other valves	
	Mitral regurgitation	
Vascular dementia	-	-

Association of FIND-AF 2.0 risk score with ischaemic stroke in patients with AF not anticoagulated

The FinACAF study is a nationwide retrospective cohort study that includes all patients documented with AF in Finland from 2004 to 2018. Patients were identified using all available national healthcare registers.

The cohort inclusion criterion was an ICD-10 diagnosis of AF and/or AFl, collectively referred to as AF, recorded between 2004 and 2018. The exclusion criteria were permanent emigration abroad before December 31, 2018, and age below 20 years at AF diagnosis. The present sub-study was conducted within a cohort of patients diagnosed with newly diagnosed AF during 2007 to 2018 that was established in previous studies of the FinACAF cohort. In this cohort, to include only patients with newly diagnosed AF, a washout period was applied by excluding those with a recorded AF diagnosis or oral anticoagulant (OAC) purchases during 2004-2006, because a medical history of less than two years was considered too short to reliably exclude the presence of a prior AF diagnosis. Additionally, those with a fulfilled OAC prescription within a year before the first AF diagnosis were excluded.

Since we aimed to assess the risk of ischaemic stroke risk when not anticoagulated, follow-up was censored when patients initiated oral anticoagulation treatment, marked by the first observed pharmacy purchase of either warfarin or a DOAC (apixaban, dabigatran, edoxaban or rivaroxaban). This method has previously been recommended for estimating event rates in untreated populations. Moreover, a patients' risk category may evolve over time due to advancing age and newly diagnosed comorbidities during a longer follow-up, usually changing from lower to higher categories. To mitigate this bias in risk group classification, we restricted the follow-up to a maximum of one year after the initial AF diagnosis. Thus, follow-up continued until the first ischaemic stroke event, the first oral anticoagulant purchase, death, end of study period on December 31st 2018, or a maximum of one years after AF diagnosis, whichever came first. Each patient's FIND-AF 2.0 score and CHA_2DS_2-VASc was calculated.

Outcome

In patients without prior ischaemic stroke, an event was considered to occur on the first date of a recorded I63 or I64 ICD-10 diagnosis code in the hospital care register. In patients with prior ischaemic stroke before or at AF diagnosis, the event was considered to occur on the date of the first new hospitalization with I63 or I64 ICD-10 code as the main diagnosis with at least a 90-day gap from the prior event, which had occurred before AF diagnosis. The I64 code of unspecified stroke was included in the outcome measure, since it has been shown that 87% of all strokes recorded with ICD-10 code of I64 are ischemic. Only ischaemic stroke diagnoses from the hospital register were included to ensure that the event of interest was truly major and clinically relevant.

Statistical analysis

Stroke rates with 95% CIs according to high and low risk categorisation were estimated with Poisson regression. The cause-specific Cox proportional hazards models accounted for oral anticoagulant initiation as a competing event, as well as for death in the ischemic stroke analyses and were compared using the non-nested likelihood ratio test. The proportional hazards assumption was tested by plotting Schoenfeld residuals with no violations observed. Statistical analyses were performed in R, version 4.0.1 (R Foundation) and statistical significance was set at $P<0.05$.

Supplemental Tables

Supplementary Table 1: Characteristics of patients in derivation and external validation cohorts stratified by outcome of AF during follow up

	Derivation		Validation							
	CPRD-GOLD (UK)		JMDC (Japan)		CHS (Israel)		CIROC (Canada)		CDARS (China)	
	No AF	AF	No AF	AF	No AF	AF	No AF	AF	No AF	AF
Participants (n)	2,073,753	7,386	7,788,729	6,515	2,162,520	4,275	617,867	10,052	148,877	268
Age (year), mean (SD)	49.8 (15.4)	73.7 (12.6)	46.1 (10.3)	55.4 (10.1)	51.0 (14.0)	78.0 (11.6)	55.0 (15.6)	70.8 (13.3)	65.3 (14.5)	75.8 (9.0)
Women (%)	1,051,942 (50.7)	3,619 (49.0)	3,994,894 (51.3)	4,747 (72.9)	1,139,309 (52.7)	2,161 (50.5)	316,326 (51.2)	4,204 (41.8)	85,659 (57.5)	157 (58.6)
Diabetes Mellitus (%)	71,966 (3.5)	815 (11.0)	667,130 (8.6)	1,525 (23.4)	340,385 (15.7)	1,842 (43.1)	42,010 (6.8)	1,254 (12.5)	96 (0.1)	0 (0.0)
Heart Failure (%)	13,717 (0.7)	650 (8.8)	129,978 (1.7)	926 (14.2)	30,029 (1.4)	472 (11.0)	7,603 (1.2)	563 (5.6)	769 (0.5)	17 (6.3)
Hypertension (%)	247,436 (11.9)	2,887 (39.1)	1,044,084 (13.4)	2,824 (43.3)	526,182 (24.3)	3,226 (75.5)	78,274 (12.7)	2,697 (26.8)	6,635 (4.5)	30 (11.2)
Stroke/TIA (%)	37,773 (1.8)	892 (12.1)	90,420 (1.2)	366 (5.6)	134,942 (6.2)	1,102 (25.8)	15,155 (2.5)	616 (6.1)	715 (0.5)	5 (1.9)
Ischaemic Heart Disease (%)	77,060 (3.7)	1,542 (20.9)	174,297 (2.2)	882 (13.5)	167,504 (7.7)	1,458 (34.1)	27,620 (4.5)	1,567 (15.6)	2,028 (1.4)	21 (7.8)
COPD (%)	24,962 (1.2)	461 (6.2)	127,942 (1.6)	276 (4.2)	56,251 (2.6)	449 (10.5)	12,846 (2.1)	655 (6.5)	652 (0.4)	9 (3.4)
Valvular Heart Disease (%)	9,497 (0.5)	376 (5.1)	39,695 (0.5)	329 (5.0)	70,246 (3.2)	722 (16.9)	4,140 (0.7)	637 (6.3)	429 (0.3)	5 (1.9)
Chronic Kidney Disease (%)	29,359 (1.4)	449 (6.1)	138,621 (1.8)	422 (6.5)	159,743 (7.4)	740 (17.3)	18,785 (3.0)	534 (5.3)	6 (0.0)	0 (0.0)
Rheumatoid Arthritis (%)	29,631 (1.4)	329 (4.5)	75,631 (1.0)	118 (1.8)	18,381 (0.8)	109 (2.5)	4,947 (0.8)	131 (1.3)	65 (0.0)	0 (0.0)
Hyperthyroidism (%)	16,147 (0.8)	155 (2.1)	142,493 (1.8)	213 (3.3)	26,694 (1.2)	101 (2.4)	1,901 (0.3)	21 (0.2)	223 (0.1)	3 (1.1)

AF, atrial fibrillation; CDARS, Clinical Data Analysis and Reporting System; CHS, Clalit Health Service; CIROC, Cardiovascular Imaging Registry of Calgary; COPD, Chronic Obstructive Pulmonary Disease; CPRD-GOLD, Clinical Practice Research Datalink; JMDC, Japan Medical Insurance Claims Database; SD, Standard deviation; TIA, Transient Ischaemic Attack; UK, United Kingdom

Supplementary Table 2: Prediction performance (AUROC) of FIND-AF 2.0, CHA_2DS_2-VASc and C_2HEST for newly diagnosed AF within six months across the included cohorts stratified by sex

		FIND-AF 2.0	CHA_2DS_2-VASc		C_2HEST	
		AUROC (95% CI)	AUROC (95% CI)		AUROC (95% CI)	
CPRD-GOLD (UK)	Overall	0.819 (0.809-0.829)	0.784 (0.773-0.794)	p<0.01	0.757 (0.744-0.770)	p<0.01
	Men	0.814 (0.804-0.824)	0.807 (0.793-0.821)	p=0.18	0.793 (0.777-0.810)	p<0.01
	Women	0.816 (0.805-0.826)	0.776 (0.760-0.793)	p<0.01	0.746 (0.727-0.765)	p<0.01
JMDC (Japan)	Overall	0.751 (0.745-0.757)	0.624 (0.617-0.633)	p<0.01	0.678 (0.672-0.684)	p<0.01
	Men	0.736 (0.714-0.757)	0.690 (0.682-0.698)	p<0.01	0.669 (0.660-0.677)	p<0.01
	Women	0.754 (0.733-0.776)	0.708 (0.694-0.722)	p<0.01	0.689 (0.675-0.703)	p<0.01
CHS (Israel)	Overall	0.835 (0.828-0.842)	0.838 (0.831-0.845)	p=0.55	0.834 (0.826-0.841)	p=0.41
	Men	0.829 (0.809-0.845)	0.810 (0.791-0.829)	p=0.16	0.820 (0.802-0.839)	p=0.94
	Women	0.856 (0.837-0.875)	0.845 (0.827-0.863)	p=0.41	0.857 (0.838-0.876)	p=0.49
CIROC (Canada)	Overall	0.747 (0.741-0.753)	0.695 (0.690-0.701)	p<0.01	0.697 (0.692-0.702)	p<0.01
	Men	0.743 (0.721-0.764)	0.712 (0.705-0.720)	p<0.01	0.673 (0.665-0.681)	p<0.01
	Women	0.792 (0.771-0.812)	0.701 (0.692-0.709)	p<0.01	0.731 (0.722-0.739)	p<0.01
CDARS (China)	Overall	0.753 (0.725-0.771)	0.709 (0.680-0.739)	p=0.02	0.682 (0.651-0.713)	p<0.01
	Men	0.719 (0.682-0.756)	0.697 (0.642-0.751)	p=0.51	0.661 (0.605-0.717)	p=0.09
	Women	0.766 (0.730-0.802)	0.749 (0.705-0.794)	p=0.56	0.698 (0.661-0.743)	p=0.01

P values compare performance of model with FIND-AF 2.0.

AUC, area under the receiver operating characteristic curve; CDARS, Clinical Data Analysis and Reporting System; CHS, Clalit Health Services; CI, confidence interval; CIROC, Cardiovascular Imaging Registry of Calgary; CPRD-GOLD, Clinical Practice Research Datalink; JMDC, Japan Medical Insurance Claims Database; UK, United Kingdom.

Supplementary Table 3: Area under the precision-recall curve (PR-AUC) of FIND-AF 2.0, CHA_2DS_2-VASc and C_2HEST for newly diagnosed AF within six months

			FIND-AF 2.0	CHA_2DS_2-VASc	C_2HEST
		AF incidence, %	PR-AUC (%), 95% CI	PR-AUC (%), 95% CI	PR-AUC (%), 95% CI
CPRD-GOLD (UK)	Overall	0.35	3.01 (2.66-3.43)	2.00 (1.78-2.23)	1.52 (1.35-1.72)
	Men	0.37	2.92 (2.59-3.31)	2.68 (2.28-3.19)	2.28 (1.91-2.78)
	Women	0.34	2.81 (2.45-3.19)	1.77 (1.51-2.13)	1.32 (1.12-1.59)
JMDC (Japan)	Overall	0.08	0.34 (0.32-0.36)	0.14 (0.13-0.15)	0.19 (0.18-0.20)
	Men	0.05	0.17 (0.14-0.20)	0.12 (0.11-0.12)	0.10 (0.10-0.11)
	Women	0.12	0.50 (0.41-0.63)	0.34 (0.30-0.38)	0.29 (0.27-0.32)
CHS (Israel)	Overall	0.20	2.17 (1.96-2.41)	2.27 (2.05-2.52)	2.14 (1.91-2.37)
	Men	0.21	2.07 (1.59-2.62)	1.61 (1.28-2.07)	1.83 (1.46-2.40)
	Women	0.19	2.88 (2.15-3.97)	2.42 (1.86-3.23)	2.93 (2.18-4.04)
CIROC (Canada)	Overall	1.60	5.84 (5.57-6.13)	3.98 (3.85-4.15)	4.04 (3.91-4.18)
	Men	1.90	6.63 (5.62-7.85)	5.28 (5.03-5.58)	4.09 (3.90-4.30)
	Women	1.31	7.26 (5.97-8.85)	3.42 (3.22-3.62)	4.27 (3.98-4.55)
CDARS (China)	Overall	0.18	0.75 (0.58-0.90)	0.51 (0.42-0.66)	0.42 (0.35-0.53)
	Men	0.18	0.54 (0.41-0.75)	0.46 (0.32-0.72)	0.36 (0.27-0.53)
	Women	0.18	0.87 (0.62-1.30)	0.73 (0.51-1.18)	0.48 (0.38-0.69)

PR-AUC, area under the precision–recall curve; CDARS, Clinical Data Analysis and Reporting System; CHS, Clalit Health Services; CI, confidence interval; CIROC, Cardiovascular Imaging Registry of Calgary; CPRD-GOLD, Clinical Practice Research Datalink; JMDC, Japan Medical Insurance Claims Database; UK, United Kingdom.

Supplementary Table 4: Comparison between prospective interventional non-randomised validation study participants and withdrawals

	Study participants (n = 1,923)	Withdrawals (n = 37)
Age (time of consent) Mean (SD)	70.2 (9.4)	68.1 (12.6)
Female (%)	958 (49.8)	20 (54.1)
White Ethnicity (%)	1,843 (95.8)	35 (94.6)
Diabetes Mellitus (%)	631 (32.8)	16 (43.2)
Heart Failure (%)	79 (4.1)	3 (8.1)
Hypertension (%)	1,606 (83.6)	24 (64.9)
Stroke or TIA (%)	177 (9.2)	9 (24.3)
Ischaemic Heart Disease (%)	277 (14.4)	5 (12.5)
COPD (%)	160 (8.3)	4 (10.9)
Valvular Heart Disease (%)	84 (4.4)	0 (0.0)
Chronic Kidney Disease (%)	335 (17.4)	5 (13.5)
Rheumatoid arthritis (%)	33 (1.7)	0 (0.0)
Hyperthyroidism (%)	14 (0.7)	0 (0.0)

COPD, Chronic Obstructive Pulmonary Disease; SD, Standard deviation; TIA, Transient Ischaemic Attack

Supplementary Table 5: Participating general practice sites in the prospective interventional non-randomised validation study

	Patients recruited (n)	Deprivation Score (IMD)	Decile of deprivation
**Affinity Care PCN**			
Shipley Medical Centre	119	32.0	7
Willow Medical Centre	394	21.7	5
Thornton Medical Centre	124	21.7	5
Sunnybank Surgery	82	32.9	7
Cowgill Surgery	27	32.9	7
Haigh Hall	158	33.7	7
**West Leeds Family PCN**			
Gleanlea Surgery	137	16.0	4
Calverley Surgery	107	16.0	4
West Lodge Surgery	320	21.3	5
**Bradford South PCN**			
Horton Park	76	49.5	9
**Dewsbury and Thornhill PCN**			
Paddock Surgery	205	28.7	6
Windsor Medical Centre	89	30.1	6
Albion Mount Medical Practice	128	25.9	6
Sidings Healthcare Centre	45	34.5	7
Heald's Road Surgery	47	37.0	8

IMD, Index of Multiple Deprivation; PCN, Primary Care Network
Interpretation of deprivation: 1 = most deprived 10% of areas in England, 10 = least deprived 10% of areas in England.

Supplementary Table 6: Sensitivity, negative and positive predictive values for newly diagnosed atrial fibrillation at different FIND-AF 2.0 score thresholds in the prospective interventional non-randomised validation study

Patient groups	AF event/total	FIND-AF 2.0 threshold							
		0.003	0.004	0.005	0.006	0.007	0.008	0.009	0.010
All patients	51/1923								
No of patients ≥ threshold, n (%)		1445 (75.2%)	1256 (65.3%)	1059 (55.1%)	864 (45.0%)	704 (36.6%)	554 (28.8%)	433 (22.5%)	348 (18.1%)
No of AF patients ≥ threshold, n (%)		49 (96.1%)	48 (94.1%)	46 (90.2%)	41 (80.4%)	33 (64.7%)	24 (47.1%)	18 (35.3%)	15 (29.4%)
Sensitivity		96.1 (86.8-98.9)	94.1 (84.1-98.0)	90.2 (79.0-95.7)	80.4 (67.5-89.0)	64.7 (51.0-76.4)	47.1 (34.1-60.5)	35.3 (23.6-49.0)	29.4 (18.7-43.0)
PPV		3.4 (2.6-4.5)	3.8 (2.9-5.0)	4.3 (3.3-5.7)	4.7 (3.5-6.4)	4.7 (3.4-6.5)	4.3 (2.9-6.4)	4.2 (2.6-6.5)	4.3 (2.6-7.0)
NPV		99.6 (98.5-99.9)	99.6 (98.7-99.8)	99.4 (98.7-99.8)	99.1 (98.3-99.5)	98.5 (97.7-99.1)	98.0 (97.1-98.6)	97.8 (96.9-98.4)	97.7 (96.9-98.3)
Adjusted NPV*		99.9 (99.8-100.0)	99.9 (99.8-100.0)	99.9 (99.8-100.0)	99.9 (99.8-99.9)	99.8 (99.7-99.9)	99.7 (99.6-99.8)	99.7 (99.6-99.8)	99.7 (99.6-99.7)
Age < 75 years	19/1242								
No of patients ≥ threshold, n (%)		784 (63.2%)	628 (50.6%)	475 (38.3%)	349 (28.1%)	261 (21.0%)	189 (15.2%)	122 (9.8%)	96 (7.7%)
No of AF patients ≥ threshold, n (%)		18 (94.7%)	17 (89.5%)	16 (84.2%)	14 (73.7%)	9 (47.4%)	8 (42.1%)	6 (31.6%)	5 (26.3%)
Sensitivity		94.7 (74.0-99.9)	89.5 (66.9-98.7)	84.2 (60.4-96.6)	73.7 (48.8-90.9)	47.4 (24.4-71.1)	42.1 (20.3-66.5)	31.6 (12.6-56.6)	26.3 (9.1-51.2)
PPV		2.3 (1.4-3.6)	2.7 (1.6-4.3)	3.4 (1.9-5.4)	4.0 (2.2-6.7)	3.5 (1.6-6.5)	4.3 (1.9-8.2)	4.9 (1.8-10.4)	5.2 (1.7-11.7)
NPV		99.8 (98.8-100.0)	99.7 (98.8-100.0)	99.6 (98.9-99.9)	99.4 (98.7-99.8)	99.0 (98.1-99.5)	99.0 (98.1-99.5)	98.8 (98.0-99.4)	98.8 (98.0-99.3)
Age >= 75 years	32/681								
No of patients ≥ threshold, n (%)		661 (97.1%)	628 (92.2%)	584 (85.8%)	515 (75.6%)	443 (65.1%)	365 (53.6%)	311 (45.7%)	252 (37.0%)
No of AF patients ≥ threshold, n (%)		31 (96.9%)	31 (96.9%)	30 (93.8%)	27 (84.4%)	24 (75.0%)	16 (50.0%)	12 (37.5%)	10 (31.2%)
Sensitivity		96.9 (83.8-99.9)	96.9 (83.8-99.9)	93.8 (79.2-99.2)	84.4 (67.2-94.7)	75.0 (56.6-88.5)	50.0 (31.9-68.1)	37.5 (21.1-56.3)	31.2 (16.1-50.0)
PPV		4.7 (3.2-6.6)	4.9 (3.4-6.9)	5.1 (3.5-7.3)	5.2 (3.5-7.5)	5.4 (3.5-8.0)	4.4 (2.5-7.0)	3.9 (2.0-6.6)	4.0 (1.9-7.2)
NPV		95.0 (75.1-99.9)	98.1 (89.9-100.0)	97.9 (92.7-99.7)	97.0 (93.1-99.0)	96.6 (93.5-98.5)	94.9 (91.9-97.1)	94.6 (91.8-96.7)	94.9 (92.3-96.8)
Men	33/965								
No of patients ≥ threshold, n (%)		652 (67.6%)	539 (55.9%)	440 (45.6%)	342 (35.4%)	242 (25.1%)	177 (18.3%)	132 (13.7%)	109 (11.3%)
No of AF patients ≥ threshold, n (%)		31 (93.9%)	30 (90.9%)	28 (84.8%)	25 (75.8%)	18 (54.5%)	12 (36.4%)	9 (27.3%)	8 (24.2%)
Sensitivity		93.9 (79.8-99.3)	90.9 (75.7-98.1)	84.8 (68.1-94.9)	75.8 (57.7-88.9)	54.5 (36.4-71.9)	36.4 (20.4-54.9)	27.3 (13.3-45.5)	24.2 (11.1-42.3)
PPV		4.8 (3.3-6.7)	5.6 (3.8-7.9)	6.4 (4.3-9.1)	7.3 (4.8-10.6)	7.4 (4.5-11.5)	6.8 (3.6-11.5)	6.8 (3.2-12.5)	7.3 (3.2-14.0)
NPV		99.4 (97.7-99.9)	99.3 (98.0-99.9)	99.0 (97.8-99.7)	98.7 (97.5-99.4)	97.9 (96.6-98.8)	97.3 (96.0-98.3)	97.1 (95.7-98.1)	97.1 (95.7-98.1)
Women	18/958								
No of patients ≥ threshold, n (%)		793 (82.9%)	717 (74.9%)	619 (64.7%)	522 (54.5%)	462 (48.3%)	377 (39.4%)	301 (31.5%)	239 (25.0%)
No of AF patients ≥ threshold, n (%)		18 (100.0%)	18 (100.0%)	18 (100.0%)	16 (88.9%)	15 (83.3%)	12 (66.7%)	9 (50.0%)	7 (38.9%)
Sensitivity		100.0 (81.5-100.0)	100.0 (81.5-100.0)	100.0 (81.5-100.0)	88.9 (65.3-98.6)	83.3 (58.6-96.4)	66.7 (41.0-86.7)	50.0 (26.0-74.0)	38.9 (17.3-64.3)

PPV		2.3 (1.4-3.6)	2.5 (1.5-3.9)	2.9 (1.7-4.6)	3.1 (1.8-4.9)	3.2 (1.8-5.3)	3.2 (1.7-5.5)	3.0 (1.4-5.6)	2.9 (1.2-5.9)
NPV		100.0 (97.8-100.0)	100.0 (98.5-100.0)	100.0 (98.9-100.0)	99.5 (98.3-99.9)	99.4 (98.2-99.9)	99.0 (97.8-99.6)	98.6 (97.4-99.4)	98.5 (97.3-99.2)
Patients with hypertension	40/1605								
No of patients ≥ threshold, n (%)		1207 (75.2%)	1048 (65.3%)	877 (54.6%)	718 (44.7%)	587 (36.6%)	467 (29.1%)	362 (22.6%)	289 (18.0%)
No of AF patients ≥ threshold, n (%)		39 (97.5%)	38 (95.0%)	37 (92.5%)	35 (87.5%)	27 (67.5%)	22 (55.0%)	16 (40.0%)	13 (32.5%)
Sensitivity		97.5 (86.8-99.9)	95.0 (83.1-99.4)	92.5 (79.6-98.4)	87.5 (73.2-95.8)	67.5 (50.9-81.4)	55.0 (38.5-70.7)	40.0 (24.9-56.7)	32.5 (18.6-49.1)
PPV		3.2 (2.3-4.4)	3.6 (2.6-4.9)	4.2 (3.0-5.8)	4.9 (3.4-6.7)	4.6 (3.1-6.6)	4.7 (3.0-7.0)	4.4 (2.5-7.1)	4.5 (2.4-7.6)
NPV		99.7 (98.6-100.0)	99.6 (98.7-100.0)	99.6 (98.8-99.9)	99.4 (98.7-99.8)	98.7 (97.8-99.3)	98.4 (97.5-99.1)	98.1 (97.1-98.8)	97.9 (97.0-98.6)
Patients without hypertension	11/318								
No of patients ≥ threshold, n (%)		238 (75.1%)	208 (65.6%)	182 (57.4%)	146 (46.1%)	117 (36.9%)	87 (27.4%)	71 (22.4%)	59 (18.6%)
No of AF patients ≥ threshold, n (%)		10 (90.9%)	10 (90.9%)	9 (81.8%)	6 (54.5%)	6 (54.5%)	2 (18.2%)	2 (18.2%)	2 (18.2%)
Sensitivity		90.9 (58.7-99.8)	90.9 (58.7-99.8)	81.8 (48.2-97.7)	54.5 (23.4-83.3)	54.5 (23.4-83.3)	18.2 (2.3-51.8)	18.2 (2.3-51.8)	18.2 (2.3-51.8)
PPV		4.2 (2.0-7.6)	4.8 (2.3-8.7)	4.9 (2.3-9.2)	4.1 (1.5-8.7)	5.1 (1.9-10.8)	2.3 (0.3-8.1)	2.8 (0.3-9.8)	3.4 (0.4-11.7)
NPV		98.7 (93.1-100.0)	99.1 (94.9-100.0)	98.5 (94.7-99.8)	97.1 (93.3-99.0)	97.5 (94.2-99.2)	96.1 (92.7-98.2)	96.3 (93.1-98.3)	96.5 (93.5-98.4)
Patients with diabetes	13/631								
No of patients ≥ threshold, n (%)		414 (65.6%)	349 (55.3%)	308 (48.8%)	255 (40.4%)	214 (33.9%)	171 (27.1%)	131 (20.8%)	112 (17.7%)
No of AF patients ≥ threshold, n (%)		12 (92.3%)	11 (84.6%)	11 (84.6%)	8 (61.5%)	6 (46.2%)	1 (7.7%)	1 (7.7%)	0 (0.0%)
Sensitivity		92.3 (64.0-99.8)	84.6 (54.6-98.1)	84.6 (54.6-98.1)	61.5 (31.6-86.1)	46.2 (19.2-74.9)	7.7 (0.2-36.0)	7.7 (0.2-36.0)	0.0 (0.0-24.7)
PPV		2.9 (1.5-5.0)	3.2 (1.6-5.6)	3.6 (1.8-6.3)	3.1 (1.4-6.1)	2.8 (1.0-6.0)	0.6 (0.0-3.2)	0.8 (0.0-4.2)	0.0 (0.0-3.2)
NPV		99.5 (97.5-100.0)	99.3 (97.5-99.9)	99.4 (97.8-99.9)	98.7 (96.9-99.6)	98.3 (96.6-99.3)	97.4 (95.5-98.6)	97.6 (95.8-98.8)	97.5 (95.8-98.7)
Patients without diabetes	38/1292								
No of patients ≥ threshold, n (%)		1031 (79.9%)	907 (70.3%)	751 (58.2%)	609 (47.2%)	490 (38.0%)	383 (29.7%)	302 (23.4%)	236 (18.3%)
No of AF patients ≥ threshold, n (%)		37 (97.4%)	37 (97.4%)	35 (92.1%)	33 (86.8%)	27 (71.1%)	23 (60.5%)	17 (44.7%)	15 (39.5%)
Sensitivity		97.4 (86.2-99.9)	97.4 (86.2-99.9)	92.1 (78.6-98.3)	86.8 (71.9-95.6)	71.1 (54.1-84.6)	60.5 (43.4-76.0)	44.7 (28.6-61.7)	39.5 (24.0-56.6)
PPV		3.6 (2.5-4.9)	4.1 (2.9-5.6)	4.7 (3.3-6.4)	5.4 (3.8-7.5)	5.5 (3.7-7.9)	6.0 (3.8-8.9)	5.6 (3.3-8.9)	6.4 (3.6-10.3)
NPV		99.6 (97.9-100.0)	99.7 (98.6-100.0)	99.4 (98.4-99.9)	99.3 (98.3-99.8)	98.6 (97.6-99.3)	98.3 (97.3-99.1)	97.9 (96.8-98.7)	97.8 (96.7-98.6)

Supplementary Table 7: Sensitivity, negative and positive predictive values for newly diagnosed atrial fibrillation at different CHA₂DS₂-VASc thresholds in the prospective interventional interventional non-randomised validation study

Patient groups	Event/total	CHA_2DS_2-VASc Threshold			
		Men ≥2<br>Women ≥3	Men ≥3<br>Women ≥4	Men ≥4<br>Women ≥5	Men ≥5<br>Women ≥6
All patients	51/1923				
No of patients ≥ threshold, n (%)		1923 (100.0%)	973 (50.6%)	342 (17.8%)	92 (4.8%)
No of AF patients ≥ threshold, n (%)		19 (100.0%)	11 (57.9%)	5 (26.3%)	0 (0.0%)
Sensitivity		100.0 (93.0-100.0)	72.5 (59.1-82.9)	23.5 (14.0-36.7)	3.9 (1.0-13.2)
PPV		2.7 (2.1-3.6)	3.8 (2.8-5.2)	3.5 (2.0-6.0)	2.1 (0.6-3.8)
NPV		100.0 (95.6-100.0)	98.5 (97.5-99.1)	97.5 (96.6-98.1)	97.3 (96.5-97.9)
Adjusted NPV*		100.0 (97.7-100.0)	99.8 (99.6-99.9)	99.6 (99.6-99.7)	99.6 (99.6-99.6)
Age < 75 years	19/1242				
No of patients ≥ threshold, n (%)		1163 (93.6%)	411 (33.1%)	116 (9.3%)	24 (1.9%)
No of AF patients ≥ threshold, n (%)		19 (100.0%)	11 (57.9%)	5 (26.3%)	0 (0.0%)
Sensitivity		100.0 (83.2-100.0)	57.9 (36.3-76.9)	26.3 (11.8-48.8)	0.0 (0.0-16.8)
PPV		1.6 (1.1-2.5)	2.7 (1.5-4.7)	4.3 (1.9-9.8)	0.0 (0.0-13.8)
NPV		100.0 (95.4-100.0)	99.0 (98.1-99.5)	98.8 (97.9-99.3)	98.4 (97.6-99.0)
Age >= 75 years	32/681				
No of patients ≥ threshold, n (%)		677 (99.4%)	562 (82.5%)	226 (33.2%)	68 (10.0%)
No of AF patients ≥ threshold, n (%)		32 (100.0%)	26 (81.2%)	7 (21.9%)	2 (6.2%)
Sensitivity		100.0 (89.3-100.0)	81.2 (64.7-91.1)	21.9 (11.0-38.8)	6.2 (1.7-20.1)
PPV		4.7 (3.4-6.6)	4.6 (3.2-6.7)	3.1 (1.5-6.3)	2.9 (0.8-10.1)
NPV		100.0 (51.0-100.0)	95.0 (89.4-97.7)	94.5 (92.0-96.3)	95.1 (93.1-96.6)
Men	33/965				
No of patients ≥ threshold, n (%)		931 (96.5%)	536 (55.5%)	200 (20.7%)	61 (6.3%)
No of AF patients ≥ threshold, n (%)		33 (100.0%)	27 (81.8%)	9 (27.3%)	2 (6.1%)
Sensitivity		100.0 (89.6-100.0)	81.8 (65.6-91.4)	27.3 (15.1-44.2)	6.1 (1.7-19.6)
PPV		3.5 (2.5-4.9)	5.0 (3.5-7.2)	4.5 (2.4-8.3)	3.3 (0.9-11.2)
NPV		100.0 (89.8-100.0)	98.6 (97.0-99.4)	96.9 (95.4-97.9)	96.6 (95.2-97.6)
Women	18/958				
No of patients ≥ threshold, n (%)		909 (94.9%)	437 (45.6%)	142 (14.8%)	31 (3.2%)
No of AF patients ≥ threshold, n (%)		18 (100.0%)	10 (55.6%)	3 (16.7%)	0 (0.0%)
Sensitivity		100.0 (82.4-100.0)	55.6 (33.7-75.4)	16.7 (5.8-39.2)	0.0 (0.0-17.6)

PPV		2.0 (1.3-3.1)	2.3 (1.2-4.2)	2.1 (0.7-6.0)	0.0 (0.0-11.0)
NPV		100.0 (92.7-100.0)	98.5 (97.0-99.2)	98.2 (97.0-98.9)	98.1 (97.0-98.8)
Patients with hypertension	40/1605				
No of patients ≥ threshold, n (%)		1549 (96.5%)	861 (53.6%)	306 (19.1%)	78 (4.9%)
No of AF patients ≥ threshold, n (%)		40 (100.0%)	31 (77.5%)	11 (27.5%)	2 (5.0%)
Sensitivity		100.0 (91.2-100.0)	77.5 (62.5-87.7)	27.5 (16.1-42.8)	5.0 (1.4-16.5)
PPV		2.6 (1.9-3.5)	3.6 (2.5-5.1)	3.6 (2.0-6.3)	2.6 (0.7-8.9)
NPV		100.0 (93.7-100.0)	98.8 (97.7-99.4)	97.8 (96.8-98.4)	97.5 (96.6-98.2)
Patients without hypertension	11/318				
No of patients ≥ threshold, n (%)		291 (91.8%)	112 (35.3%)	36 (11.4%)	14 (4.4%)
No of AF patients ≥ threshold, n (%)		11 (100.0%)	6 (54.5%)	1 (9.1%)	0 (0.0%)
Sensitivity		100.0 (74.1-100.0)	54.5 (28.0-78.7)	9.1 (1.6-37.7)	0.0 (0.0-25.9)
PPV		3.8 (2.1-6.7)	5.4 (2.5-11.2)	2.8 (0.5-14.2)	0.0 (0.0-21.5)
NPV		100.0 (87.1-100.0)	97.5 (94.4-98.9)	96.4 (93.6-98.0)	96.4 (93.6-98.0)
Patients with diabetes	13/631				
No of patients ≥ threshold, n (%)		620 (98.3%)	389 (61.6%)	185 (29.3%)	42 (6.7%)
No of AF patients ≥ threshold, n (%)		13 (100.0%)	11 (84.6%)	7 (53.8%)	0 (0.0%)
Sensitivity		100.0 (77.2-100.0)	84.6 (57.8-95.7)	53.8 (29.1-76.8)	0.0 (0.0-22.8)
PPV		2.1 (1.2-3.6)	2.8 (1.6-5.0)	3.8 (1.8-7.6)	0.0 (0.0-8.4)
NPV		100.0 (74.1-100.0)	99.2 (97.0-99.8)	98.7 (97.1-99.4)	97.8 (96.3-98.7)
Patients without diabetes	38/1292				
No of patients ≥ threshold, n (%)		1220 (94.4%)	584 (45.2%)	157 (12.2%)	50 (3.9%)
No of AF patients ≥ threshold, n (%)		38 (100.0%)	26 (68.4%)	5 (13.2%)	2 (5.3%)
Sensitivity		100.0 (90.8-100.0)	68.4 (52.5-80.9)	13.2 (5.8-27.3)	5.3 (1.5-17.3)
PPV		3.1 (2.3-4.2)	4.5 (3.1-6.4)	3.2 (1.4-7.2)	4.0 (1.1-13.5)
NPV		100.0 (94.9-100.0)	98.3 (97.1-99.0)	97.1 (95.9-97.9)	97.1 (96.0-97.9)

*Negative predictive values for all patients were adjusted to a target AF prevalence of 0.4% to illustrate expected performance in a general population setting.

Supplementary Table 8: Sensitivity, negative and positive predictive values for newly diagnosed atrial fibrillation diagnosis at different C_2HEST thresholds in the prospective interventional non-randomised validation study

Patient groups	Event/total	C_2HEST Threshold				
		≥1	≥2	≥3	≥4	≥5
All patients	51/1923					
No of patients ≥ threshold, n (%)		1830 (95.2%)	934 (48.6%)	626 (32.6%)	133 (6.9%)	28 (1.5%)
No of AF patients ≥ threshold, n (%)		49 (96.1%)	35 (68.6%)	28 (54.9%)	6 (11.8%)	1 (2.0%)
Sensitivity		96.1 (86.7-98.9)	68.6 (55.0-79.7)	54.9 (41.3-67.8)	11.8 (5.0-23.4)	1.9 (0.3-10.3)
PPV		2.7 (2.0-3.5)	3.7 (2.7-5.1)	4.5 (3.1-6.3)	4.4 (0.6-7.5)	3.3 (0.6-16.7)
NPV		97.8 (92.4-99.4)	98.4 (97.4-99.0)	98.2 (97.3-98.8)	97.5 (96.5-98.0)	97.4 (96.5-98.0)
Adjusted NPV*		99.7 (98.6-100.0)	99.8 (99.6-99.9)	99.7 (99.6-99.8)	99.6 (99.6-99.7)	99.6 (99.6-99.6)
Age < 75 years	19/1242					
No of patients ≥ threshold, n (%)		1149 (92.5%)	253 (20.4%)	64 (5.2%)	16 (1.3%)	3 (0.2%)
No of AF patients ≥ threshold, n (%)		17 (89.5%)	3 (15.8%)	2 (10.5%)	0 (0.0%)	0 (0.0%)
Sensitivity		89.5 (68.6-97.1)	15.8 (5.5-37.6)	10.5 (2.9-31.4)	0.0 (0.0-16.8)	0.0 (0.0-16.8)
PPV		1.5 (0.9-2.4)	1.2 (0.4-3.4)	3.0 (0.8-10.2)	0.0 (0.0-19.4)	0.0 (0.0-56.1)
NPV		97.8 (92.4-99.4)	98.4 (97.4-99.0)	98.6 (97.7-99.1)	98.4 (97.6-99.0)	98.5 (97.6-99.0)
Age >= 75 years	32/681					
No of patients ≥ threshold, n (%)		681 (100.0%)	681 (100.0%)	562 (82.5%)	117 (17.2%)	25 (3.7%)
No of AF patients ≥ threshold, n (%)		32 (100.0%)	32 (100.0%)	26 (81.2%)	6 (18.8%)	1 (3.1%)
Sensitivity		100.0 (89.3-100.0)	100.0 (89.3-100.0)	81.2 (64.7-91.1)	18.8 (8.9-35.3)	3.1 (0.6-15.7)
PPV		4.7 (3.3-6.6)	4.7 (3.3-6.6)	4.6 (3.2-6.7)	5.0 (2.3-10.4)	3.7 (0.7-18.3)
NPV		-	-	95.0 (89.4-97.7)	95.4 (93.3-96.8)	95.3 (93.4-96.6)
Men	33/965					
No of patients ≥ threshold, n (%)		918 (95.1%)	474 (49.1%)	311 (32.2%)	89 (9.2%)	17 (1.8%)
No of AF patients ≥ threshold, n (%)		32 (97.0%)	22 (66.7%)	18 (54.5%)	5 (15.2%)	1 (3.0%)
Sensitivity		97.0 (84.7-99.5)	66.7 (49.6-80.2)	54.5 (38.0-70.2)	15.2 (6.7-30.9)	3.0 (0.5-15.3)
PPV		3.5 (2.5-4.9)	4.6 (3.1-6.9)	5.8 (3.7-9.0)	5.6 (2.4-12.4)	5.6 (1.0-25.8)
NPV		97.9 (88.9-99.6)	97.8 (96.0-98.7)	97.7 (96.3-98.6)	96.8 (95.4-97.8)	96.6 (95.3-97.6)
Women	18/958					
No of patients ≥ threshold, n (%)		912 (95.2%)	460 (48.0%)	315 (32.9%)	44 (4.6%)	11 (1.1%)
No of AF patients ≥ threshold, n (%)		17 (94.4%)	13 (72.2%)	10 (55.6%)	1 (5.6%)	0 (0.0%)
Sensitivity		94.4 (74.2-99.0)	72.2 (49.1-87.5)	55.6 (33.7-75.4)	5.6 (1.0-25.8)	0.0 (0.0-17.6)
PPV		1.9 (1.2-3.0)	2.8 (1.7-4.8)	3.1 (1.7-5.7)	2.1 (0.4-11.1)	0.0 (0.0-24.2)

NPV		97.8 (88.4-99.6)	99.0 (97.7-99.6)	98.8 (97.6-99.4)	98.1 (97.0-98.8)	98.1 (97.0-98.8)
Patients with hypertension	40/1605					
No of patients ≥ threshold, n (%)		1605 (100.0%)	752 (46.8%)	570 (35.5%)	120 (7.5%)	24 (1.5%)
No of AF patients ≥ threshold, n (%)		40 (100.0%)	27 (67.5%)	26 (65.0%)	5 (12.5%)	1 (2.5%)
Sensitivity		100.0 (91.2-100.0)	67.5 (52.0-79.9)	65.0 (49.5-77.9)	12.5 (5.5-26.1)	2.5 (0.4-12.9)
PPV		2.5 (1.8-3.4)	3.6 (2.5-5.1)	4.5 (3.1-6.6)	4.1 (1.7-9.2)	3.8 (0.7-18.9)
NPV		-	98.5 (97.4-99.1)	98.6 (97.7-99.2)	97.6 (96.7-98.3)	97.5 (96.6-98.2)
Patients without hypertension	11/318					
No of patients ≥ threshold, n (%)		224 (70.7%)	182 (57.4%)	56 (17.7%)	13 (4.1%)	4 (1.3%)
No of AF patients ≥ threshold, n (%)		9 (81.8%)	8 (72.7%)	2 (18.2%)	1 (9.1%)	0 (0.0%)
Sensitivity		81.8 (52.3-94.9)	72.7 (43.4-90.3)	18.2 (5.1-47.7)	9.1 (1.6-37.7)	0.0 (0.0-25.9)
PPV		4.0 (2.1-7.4)	4.4 (2.2-8.4)	3.6 (1.0-12.1)	7.1 (1.3-31.5)	0.0 (0.0-49.0)
NPV		97.8 (92.3-99.4)	97.8 (93.6-99.2)	96.5 (93.6-98.2)	96.7 (94.0-98.2)	96.5 (93.8-98.0)
Patients with diabetes	13/631					
No of patients ≥ threshold, n (%)		590 (93.5%)	238 (37.7%)	156 (24.7%)	40 (6.3%)	11 (1.7%)
No of AF patients ≥ threshold, n (%)		12 (92.3%)	7 (53.8%)	5 (38.5%)	1 (7.7%)	0 (0.0%)
Sensitivity		92.3 (66.7-98.6)	53.8 (29.1-76.8)	38.5 (17.7-64.5)	7.7 (1.4-33.3)	0.0 (0.0-22.8)
PPV		2.0 (1.2-3.5)	2.9 (1.4-5.9)	3.2 (1.4-7.3)	2.4 (0.4-12.6)	0.0 (0.0-24.2)
NPV		97.6 (87.4-99.6)	98.5 (96.7-99.3)	98.3 (96.7-99.1)	98.0 (96.5-98.8)	97.9 (96.4-98.8)
Patients without diabetes	38/1292					
No of patients ≥ threshold, n (%)		1240 (96.0%)	696 (53.9%)	470 (36.4%)	93 (7.2%)	17 (1.3%)
No of AF patients ≥ threshold, n (%)		37 (97.4%)	28 (73.7%)	23 (60.5%)	5 (13.2%)	1 (2.6%)
Sensitivity		97.4 (86.5-99.5)	73.7 (58.0-85.0)	60.5 (44.7-74.4)	13.2 (5.8-27.3)	2.6 (0.5-13.5)
PPV		3.0 (2.2-4.1)	4.0 (2.8-5.7)	4.9 (3.3-7.2)	5.2 (2.2-11.6)	5.6 (1.0-25.8)
NPV		98.0 (89.7-99.7)	98.3 (96.9-99.1)	98.2 (97.0-98.9)	97.2 (96.2-98.0)	97.1 (96.0-97.9)

*Negative predictive values for all patients were adjusted to a target AF prevalence of 0.4% to illustrate expected performance in a general population setting.

Supplementary Table 9: Sensitivity, negative and positive predictive values for newly diagnosed atrial fibrillation at different CHARGE-AF score thresholds in the prospective interventional non-randomised validation study

Patient groups		CHARGE-AF threshold							
	Event/total	0.05	0.07	0.09	0.11	0.13	0.15	0.17	0.19
All patients (complete data)	12/957								
No of patients ≥ threshold, n (%)		830 (86.7%)	751 (78.5%)	652 (68.1%)	571 (59.7%)	496 (51.8%)	431 (45.0%)	356 (37.2%)	308 (32.2%)
No of AF patients ≥ threshold, n (%)		12 (100.0%)	12 (100.0%)	11 (91.7%)	11 (91.7%)	10 (83.3%)	10 (83.3%)	9 (75.0%)	8 (66.7%)
Sensitivity		100.0 (73.5-100.0)	100.0 (73.5-100.0)	91.7 (61.5-99.8)	91.7 (61.5-99.8)	83.3 (51.6-97.9)	83.3 (51.6-97.9)	75.0 (42.8-94.5)	66.7 (34.9-90.1)
PPV		1.4 (0.7-2.5)	1.6 (0.8-2.8)	1.7 (0.8-3.0)	1.9 (1.0-3.4)	2.0 (1.0-3.7)	2.3 (1.1-4.2)	2.5 (1.2-4.7)	2.6 (1.1-5.1)
NPV		100.0 (97.1-100.0)	100.0 (98.2-100.0)	99.7 (98.2-100.0)	99.7 (98.6-100.0)	99.6 (98.4-99.9)	99.6 (98.6-100.0)	99.5 (98.5-99.9)	99.4 (98.4-99.8)
Adjusted NPV*		100.0 (98.8-100.0)	100.0 (99.5-100.0)	99.9 (99.5-100.0)	99.9 (99.6-100.0)	99.9 (99.6-100.0)	99.9 (99.6-100.0)	99.8 (99.6-100.0)	99.8 (99.6-99.9)
All patients (imputed data)	51/1923								
No of patients ≥ threshold, n (%)		1662 (86.4%)	1507 (78.4%)	1323 (68.8%)	1161 (60.4%)	1007 (52.4%)	862 (44.8%)	707 (36.8%)	594 (30.9%)
No of AF patients ≥ threshold, n (%)		51 (100.0%)	51 (100.0%)	48 (94.1%)	43 (84.3%)	42 (82.4%)	35 (68.6%)	33 (64.7%)	29 (56.9%)
Sensitivity		100.0 (93.0-100.0)	100.0 (93.0-100.0)	94.1 (83.8-98.8)	84.3 (71.4-93.0)	82.4 (69.1-91.6)	68.6 (54.1-80.9)	64.7 (50.1-77.6)	56.9 (42.2-70.7)
PPV		3.1 (2.3-4.0)	3.4 (2.5-4.4)	3.6 (2.7-4.8)	3.7 (2.7-5.0)	4.2 (3.0-5.6)	4.1 (2.8-5.6)	4.7 (3.2-6.5)	4.9 (3.3-6.9)
NPV		100.0 (98.6-100.0)	100.0 (99.1-100.0)	99.5 (98.5-99.9)	99.0 (97.9-99.5)	99.0 (98.1-99.5)	98.5 (97.6-99.1)	98.5 (97.7-99.1)	98.3 (97.5-99.0)
Adjusted NPV*		100.0 (99.8-100.0)	100.0 (99.9-100.0)	99.9 (99.8-100.0)	99.8 (99.7-99.9)	99.9 (99.7-99.9)	99.8 (99.7-99.9)	99.8 (99.7-99.9)	99.8 (99.7-99.8)
Age < 75 years	19/1242								
No of patients ≥ threshold, n (%)		981 (79.0%)	827 (66.6%)	650 (52.3%)	501 (40.3%)	372 (30.0%)	268 (21.6%)	178 (14.3%)	132 (10.6%)
No of AF patients ≥ threshold, n (%)		19 (100.0%)	19 (100.0%)	16 (84.2%)	11 (57.9%)	11 (57.9%)	7 (36.8%)	6 (31.6%)	4 (21.1%)
Sensitivity		100.0 (82.4-100.0)	100.0 (82.4-100.0)	84.2 (60.4-96.6)	57.9 (33.5-79.7)	57.9 (33.5-79.7)	36.8 (16.3-61.6)	31.6 (12.6-56.6)	21.1 (6.1-45.6)
PPV		1.9 (1.2-3.0)	2.3 (1.4-3.6)	2.5 (1.4-4.0)	2.2 (1.1-3.9)	3.0 (1.5-5.2)	2.6 (1.1-5.3)	3.4 (1.2-7.2)	3.0 (0.8-7.6)
NPV		100.0 (98.6-100.0)	100.0 (99.1-100.0)	99.5 (98.5-99.9)	98.9 (97.9-99.5)	99.1 (98.2-99.6)	98.8 (97.9-99.4)	98.8 (97.9-99.3)	98.6 (97.8-99.2)
Age >= 75 years	32/681								
No of patients ≥ threshold, n (%)		681 (100.0%)	680 (99.9%)	673 (98.8%)	660 (96.9%)	635 (93.2%)	594 (87.2%)	529 (77.7%)	462 (67.8%)
No of AF patients ≥ threshold, n (%)		33 (100.0%)	33 (100.0%)	31 (93.9%)	28 (84.8%)	28 (84.8%)	25 (75.8%)	23 (69.7%)	19 (57.6%)
Sensitivity		100.0 (89.1-100.0)	100.0 (89.1-100.0)	100.0 (89.1-100.0)	100.0 (89.1-100.0)	96.9 (83.8-99.9)	87.5 (71.0-96.5)	84.4 (67.2-94.7)	78.1 (60.0-90.7)
PPV		4.7 (3.2-6.6)	4.7 (3.2-6.6)	4.8 (3.3-6.6)	4.8 (3.3-6.8)	4.9 (3.3-6.9)	4.7 (3.2-6.7)	5.1 (3.4-7.3)	5.4 (3.5-7.9)
NPV			100.0 (2.5-100.0)	100.0 (63.1-100.0)	100.0 (83.9-100.0)	97.8 (88.5-99.9)	95.4 (88.6-98.7)	96.7 (92.5-98.9)	96.8 (93.5-98.7)
Men	33/965								
No of patients ≥ threshold, n (%)		848 (87.9%)	790 (81.9%)	715 (74.1%)	637 (66.0%)	556 (57.6%)	492 (51.0%)	416 (43.1%)	354 (36.7%)

No of AF patients ≥ threshold, n (%)		33 (100.0%)	33 (100.0%)	31 (93.9%)	28 (84.8%)	28 (84.8%)	25 (75.8%)	23 (69.7%)	19 (57.6%)
Sensitivity		100.0 (89.4-100.0)	100.0 (89.4-100.0)	93.9 (79.8-99.3)	84.8 (68.1-94.9)	84.8 (68.1-94.9)	75.8 (57.7-88.9)	69.7 (51.3-84.4)	57.6 (39.2-74.5)
PPV		3.9 (2.7-5.4)	4.2 (2.9-5.8)	4.3 (3.0-6.1)	4.4 (2.9-6.3)	5.0 (3.4-7.2)	5.1 (3.3-7.4)	5.5 (3.5-8.2)	5.4 (3.3-8.3)
NPV		100.0 (96.9-100.0)	100.0 (97.9-100.0)	99.2 (97.1-99.9)	98.5 (96.5-99.5)	98.8 (97.2-99.6)	98.3 (96.7-99.3)	98.2 (96.7-99.1)	97.7 (96.2-98.7)
Women	18/958								
No of patients ≥ threshold, n (%)		814 (85.0%)	717 (74.8%)	608 (63.5%)	524 (54.7%)	451 (47.1%)	370 (38.6%)	291 (30.4%)	240 (25.1%)
No of AF patients ≥ threshold, n (%)		18 (100.0%)	18 (100.0%)	17 (94.4%)	15 (83.3%)	14 (77.8%)	10 (55.6%)	10 (55.6%)	10 (55.6%)
Sensitivity		100.0 (81.5-100.0)	100.0 (81.5-100.0)	94.4 (72.7-99.9)	83.3 (58.6-96.4)	77.8 (52.4-93.6)	55.6 (30.8-78.5)	55.6 (30.8-78.5)	55.6 (30.8-78.5)
PPV		2.2 (1.3-3.5)	2.5 (1.5-3.9)	2.8 (1.6-4.4)	2.9 (1.6-4.7)	3.1 (1.7-5.2)	2.7 (1.3-4.9)	3.4 (1.7-6.2)	4.2 (2.0-7.5)
NPV		100.0 (97.5-100.0)	100.0 (98.5-100.0)	99.7 (98.4-100.0)	99.3 (98.0-99.9)	99.2 (98.0-99.8)	98.6 (97.3-99.4)	98.8 (97.7-99.5)	98.9 (97.8-99.5)
Patients with hypertension	40/1605								
No of patients ≥ threshold, n (%)		1402 (87.3%)	1266 (78.8%)	1107 (68.9%)	972 (60.5%)	846 (52.7%)	724 (45.1%)	612 (38.1%)	519 (32.3%)
No of AF patients ≥ threshold, n (%)		40 (100.0%)	40 (100.0%)	38 (95.0%)	35 (87.5%)	34 (85.0%)	30 (75.0%)	28 (70.0%)	25 (62.5%)
Sensitivity		100.0 (91.2-100.0)	100.0 (91.2-100.0)	95.0 (83.1-99.4)	87.5 (73.2-95.8)	85.0 (70.2-94.3)	75.0 (58.8-87.3)	70.0 (53.5-83.4)	62.5 (45.8-77.3)
PPV		2.9 (2.0-3.9)	3.2 (2.3-4.3)	3.4 (2.4-4.7)	3.6 (2.5-5.0)	4.0 (2.8-5.6)	4.1 (2.8-5.9)	4.6 (3.1-6.5)	4.8 (3.1-7.0)
NPV		100.0 (98.2-100.0)	100.0 (98.9-100.0)	99.6 (98.6-100.0)	99.2 (98.2-99.7)	99.2 (98.3-99.7)	98.9 (97.9-99.5)	98.8 (97.9-99.4)	98.6 (97.7-99.2)
Patients without hypertension	11/318								
No of patients ≥ threshold, n (%)		260 (82.0%)	241 (76.0%)	216 (68.1%)	189 (59.6%)	161 (50.8%)	138 (43.5%)	95 (30.0%)	75 (23.7%)
No of AF patients ≥ threshold, n (%)		11 (100.0%)	11 (100.0%)	10 (90.9%)	8 (72.7%)	8 (72.7%)	5 (45.5%)	5 (45.5%)	4 (36.4%)
Sensitivity		100.0 (71.5-100.0)	100.0 (71.5-100.0)	90.9 (58.7-99.8)	72.7 (39.0-94.0)	72.7 (39.0-94.0)	45.5 (16.7-76.6)	45.5 (16.7-76.6)	36.4 (10.9-69.2)
PPV		4.2 (2.1-7.4)	4.6 (2.3-8.0)	4.6 (2.2-8.3)	4.2 (1.8-8.2)	5.0 (2.2-9.6)	3.6 (1.2-8.3)	5.3 (1.7-11.9)	5.3 (1.5-13.1)
NPV		100.0 (93.7-100.0)	100.0 (95.3-100.0)	99.0 (94.6-100.0)	97.7 (93.3-99.5)	98.1 (94.5-99.6)	96.6 (92.8-98.8)	97.3 (94.2-99.0)	97.1 (94.1-98.8)
Patients with diabetes	13/631								
No of patients ≥ threshold, n (%)		495 (78.4%)	428 (67.8%)	378 (59.9%)	337 (53.4%)	304 (48.2%)	271 (42.9%)	232 (36.8%)	211 (33.4%)
No of AF patients ≥ threshold, n (%)		13 (100.0%)	13 (100.0%)	10 (76.9%)	9 (69.2%)	9 (69.2%)	9 (69.2%)	9 (69.2%)	7 (53.8%)
Sensitivity		100.0 (75.3-100.0)	100.0 (75.3-100.0)	76.9 (46.2-95.0)	69.2 (38.6-90.9)	69.2 (38.6-90.9)	69.2 (38.6-90.9)	69.2 (38.6-90.9)	53.8 (25.1-80.8)
PPV		2.6 (1.4-4.4)	3.0 (1.6-5.1)	2.6 (1.3-4.8)	2.7 (1.2-5.0)	3.0 (1.4-5.5)	3.3 (1.5-6.2)	3.9 (1.8-7.2)	3.3 (1.3-6.7)
NPV		100.0 (97.3-100.0)	100.0 (98.2-100.0)	98.8 (96.6-99.8)	98.6 (96.6-99.6)	98.8 (96.9-99.7)	98.9 (97.2-99.7)	99.0 (97.5-99.7)	98.6 (96.9-99.5)
Patients without diabetes	38/1292								
No of patients ≥ threshold, n (%)		1167 (90.3%)	1079 (83.5%)	945 (73.1%)	824 (63.8%)	703 (54.4%)	591 (45.7%)	475 (36.8%)	383 (29.6%)
No of AF patients ≥ threshold, n (%)		38 (100.0%)	38 (100.0%)	38 (100.0%)	34 (89.5%)	33 (86.8%)	26 (68.4%)	24 (63.2%)	22 (57.9%)

Sensitivity		100.0 (90.7-100.0)	100.0 (90.7-100.0)	100.0 (90.7-100.0)	89.5 (75.2-97.1)	86.8 (71.9-95.6)	68.4 (51.3-82.5)	63.2 (46.0-78.2)	57.9 (40.8-73.7)
PPV		3.3 (2.3-4.4)	3.5 (2.5-4.8)	4.0 (2.9-5.5)	4.1 (2.9-5.7)	4.7 (3.3-6.5)	4.4 (2.9-6.4)	5.1 (3.3-7.4)	5.7 (3.6-8.6)
NPV		100.0 (97.1-100.0)	100.0 (98.3-100.0)	100.0 (98.9-100.0)	99.1 (97.8-99.8)	99.2 (98.0-99.7)	98.3 (97.0-99.1)	98.3 (97.1-99.1)	98.2 (97.2-99.0)

*Negative predictive values were adjusted to a target AF prevalence of 0.4% to illustrate expected performance in a general population setting.

Supplementary Table 10: Sensitivity, negative and positive predictive values for newly diagnosed atrial fibrillation diagnosis using age in the prospective interventional non-randomised validation study

Age Threshold (years)	75 and 76
No. of patients meet screening criteria, n (%)	231 (12.0%)
No. of AF patients meet screening criteria, n (%)	7 (13.7%)
Sensitivity	13.7 (0.7-25.7)
Positive predictive value	3.0 (1.5-6.1)
Negative predictive value	97.4 (96.5-98.1)
Adjusted NPV	99.6 (99.6-99.7)

*Age thresholds of ≥65 years and ≥70 years were not modelled as eligibility for the trial required CHA_2DS_2-VASc Men ≥2 Women ≥3, which means for patients aged <75 years they would require at least one other stroke risk factor. Thus patients aged <75 years in the trial do not reflect an all-comer population.

Supplementary Table 11: Confusion matrix summary for the different enrichment approaches in the prospective study.

	True positive	False positive	False negative	True negative	Precision	Recall	F1
FIND-AF 2.0 ≥ 0.0053	46	975	5	897	0.0451	0.9020	0.0858
CHA_2DS_2-VASc ≥2 in men and ≥3 in women	51	1789	0	83	0.0277	1.0000	0.0539
CHA_2DS_2-VASc ≥3 in men and ≥4 in women	37	936	14	936	0.0380	0.7255	0.0723
C_2HEST ≥1	49	1782	2	90	0.0267	0.9608	0.0512
C_2HEST ≥3	28	601	23	1271	0.0445	0.5490	0.0824
CHARGE-AF ≥ 0.11 (complete data)	11	560	1	385	0.0193	0.9167	0.0377
CHARGE-AF ≥ 0.11 (imputed data)	43	1118	8	754	0.0370	0.8431	0.0710
Age 75-76 years	7	224	44	1648	0.0303	0.1373	0.0470

Supplemental Figures

Supplementary Figure 1: Flow chart of process for selecting variables for the FIND-AF prediction model

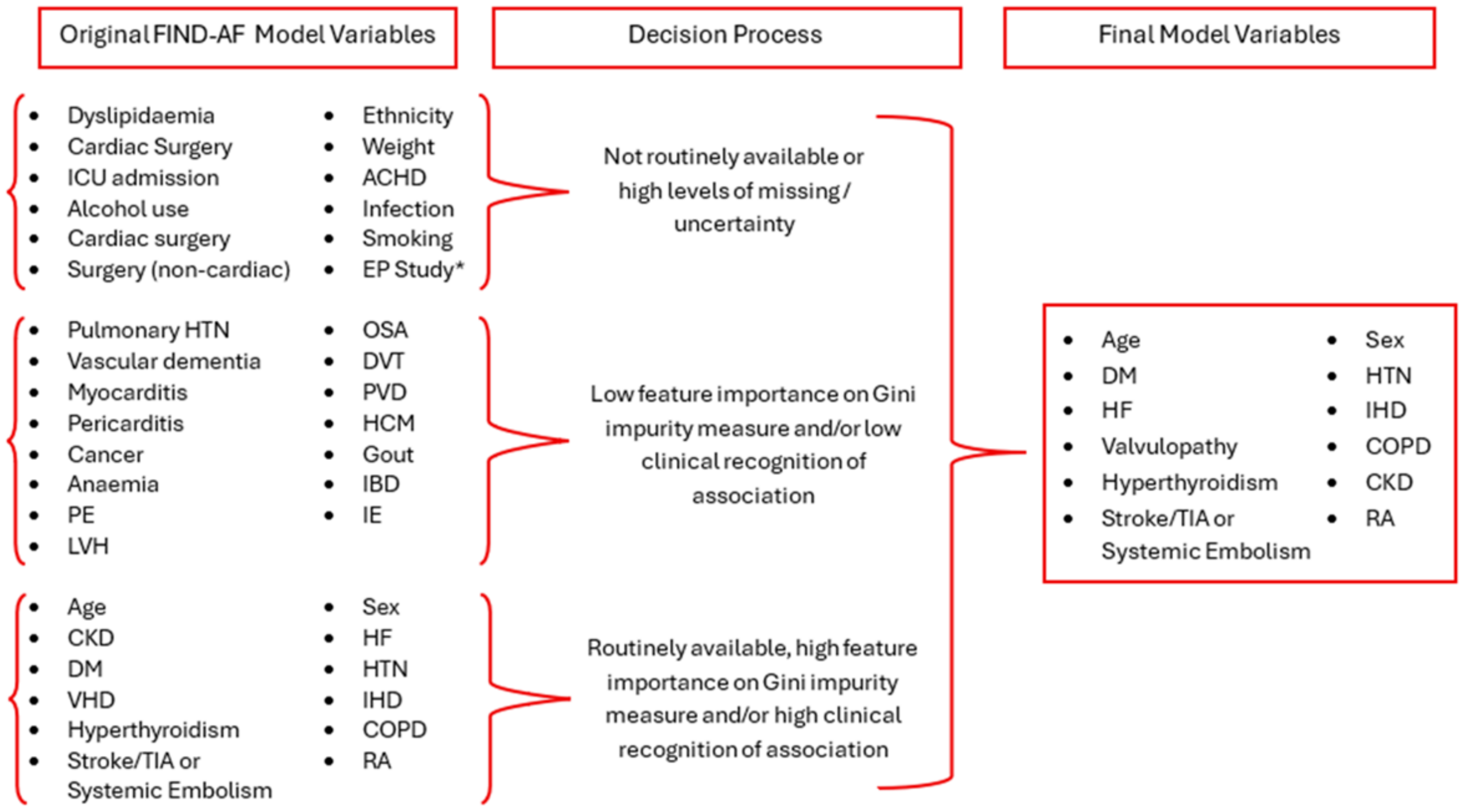

* Electrophysiology procedure affecting the atria

ACHD, adult congenital heart disease; CKD, chronic kidney disease; COPD, chronic obstructive pulmonary disease; DM, diabetes mellitus; DVT, deep vein thrombosis; EP, electrophysiology; HCM, hypertrophic cardiomyopathy; HF, heart failure; HTN, hypertension; IBD, inflammatory bowel disease; ICU, intensive care unit; IE, infective

endocarditis; IHD, ischaemic heart disease; LVH, left ventricular hypertrophy; OSA, obstructive sleep apnoea; PE, pulmonary embolism; RA, rheumatoid arthritis; VHD, valvular heart disease

Supplementary Figure 2: Variable importance in the FIND-AF prediction model quantified by mean decrease in Gini impurity

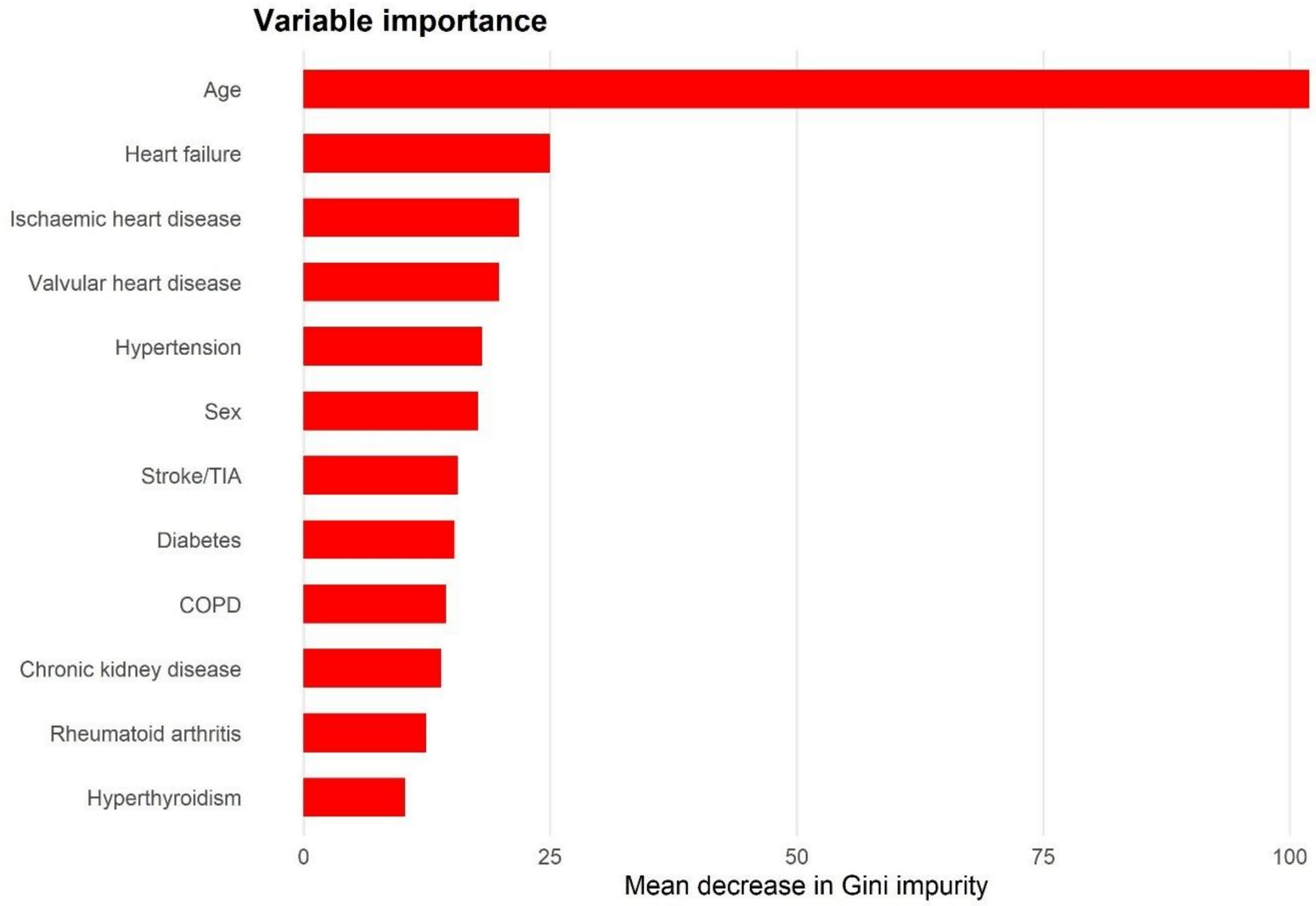

TIA, Transient Ischaemic Attack; COPD, Chronic Obstructive Pulmonary Disease

Supplementary Figure 3: Receiver operating characteristic curve for the prediction of newly diagnosed atrial fibrillation diagnosis within the next six months for FIND-AF, CHA_2DS_2-VASc, and C_2HEST across included cohorts

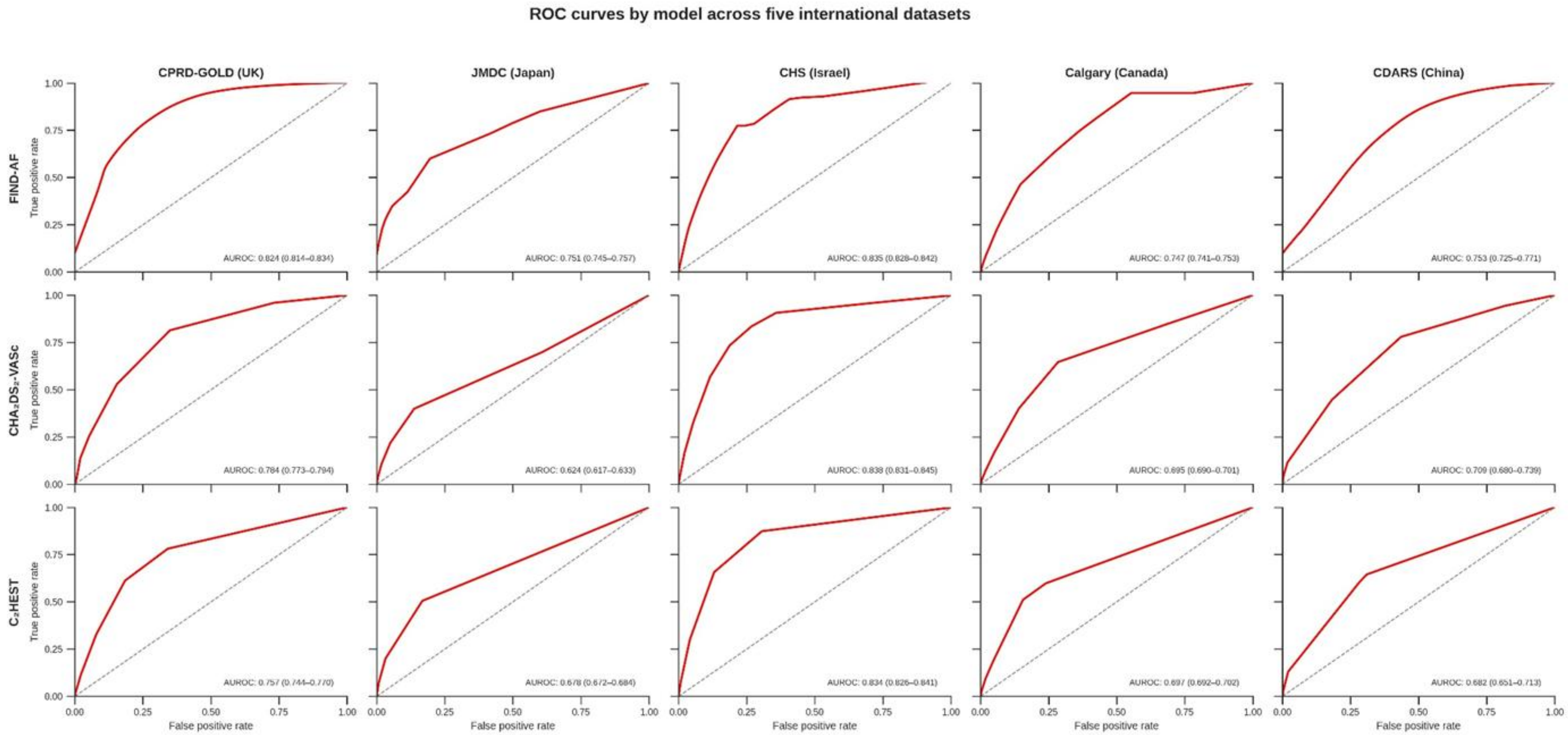

AUROC, area under the receiver operating characteristic curve; CDARS, Clinical Data Analysis and Reporting System; CHS, Clalit Health Service; CPRD, Clinical Practice Research Datalink; JMDC, Japan Medical Insurance Claims Database; ROC, Receiver Operating Characteristic; UK, United Kingdom

Supplementary Figure 4. Prospective interventional non-randomised validation study flowchart

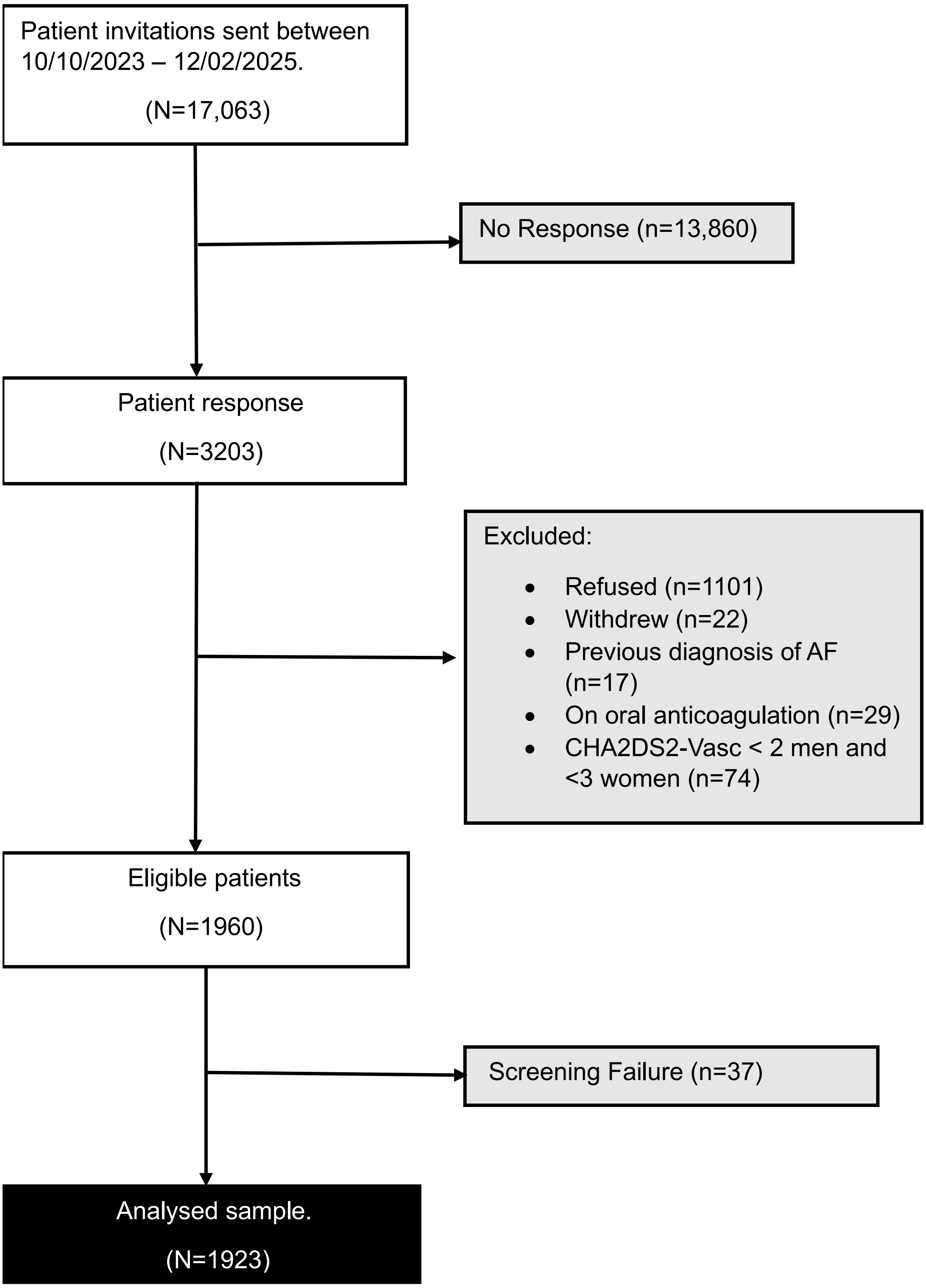

AF, Atrial Fibrillation

Supplementary Figure 5: Distribution of FIND-AF 2.0 score across prospective interventional non-randomised validation study participants stratified by high and low risk

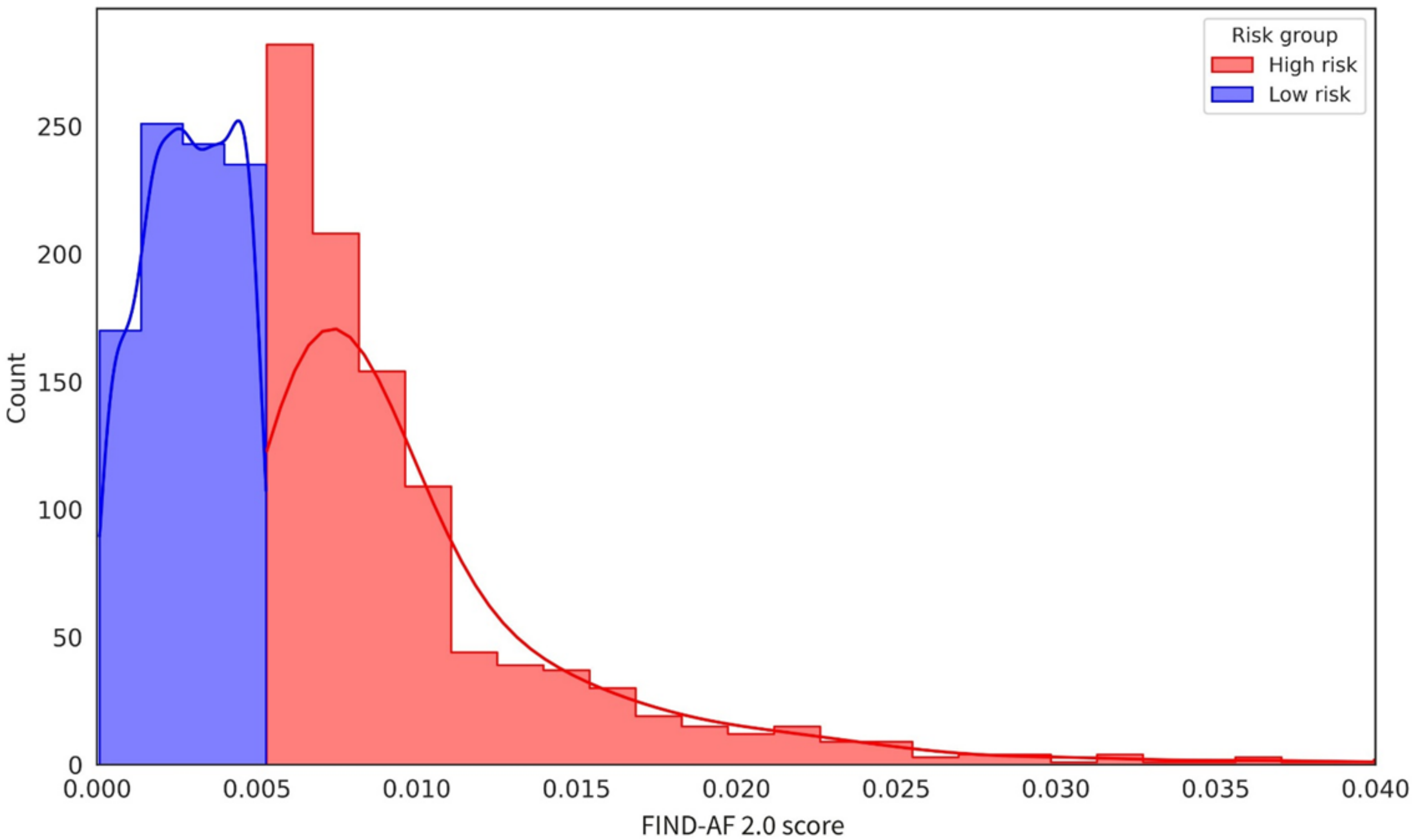

Supplementary Figure 6: Distribution of number of ECG recordings taken per participant stratified by high and low risk

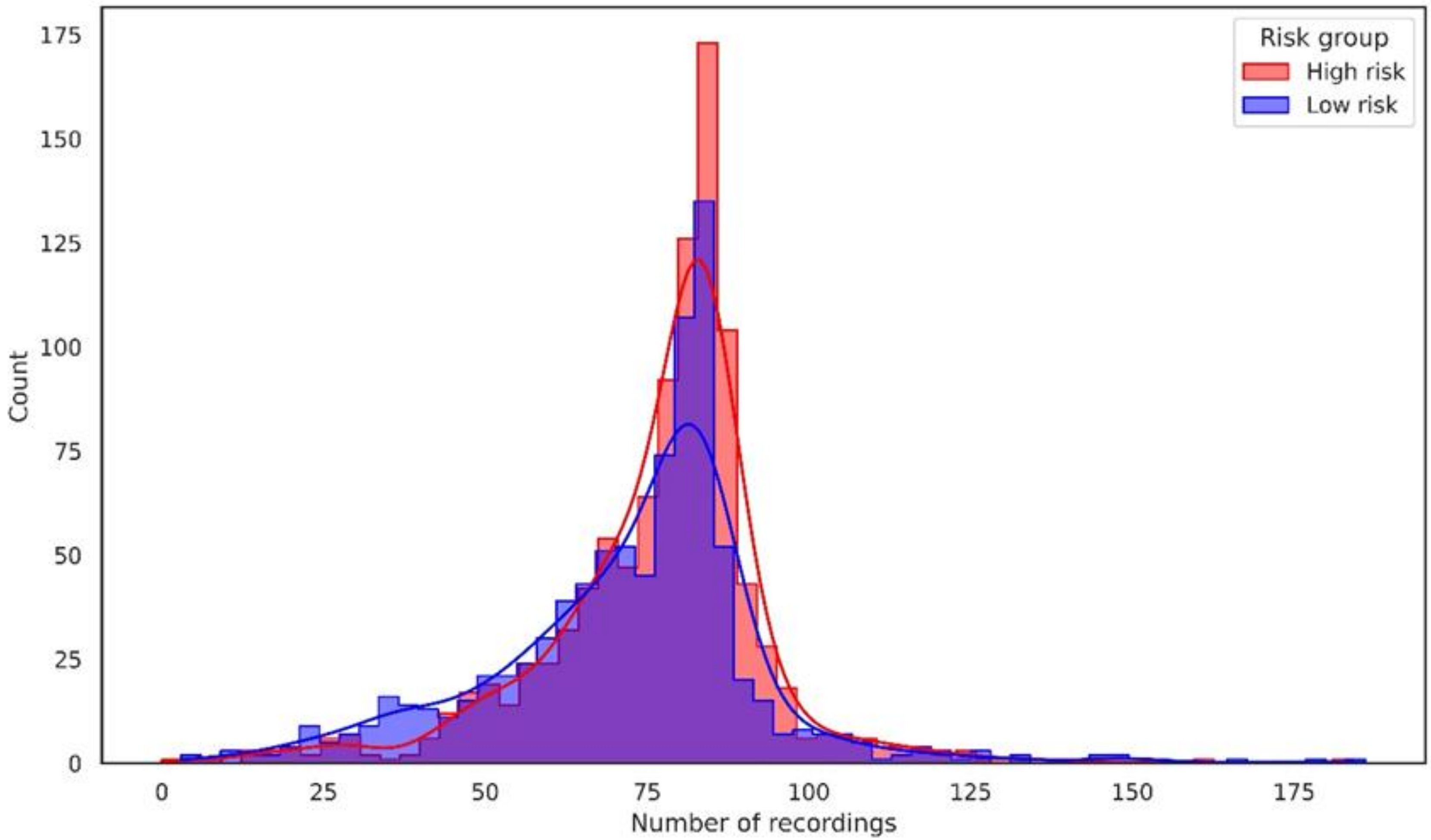

Supplementary Figure 7: Percentage of recordings with AF amongst detected AF cases

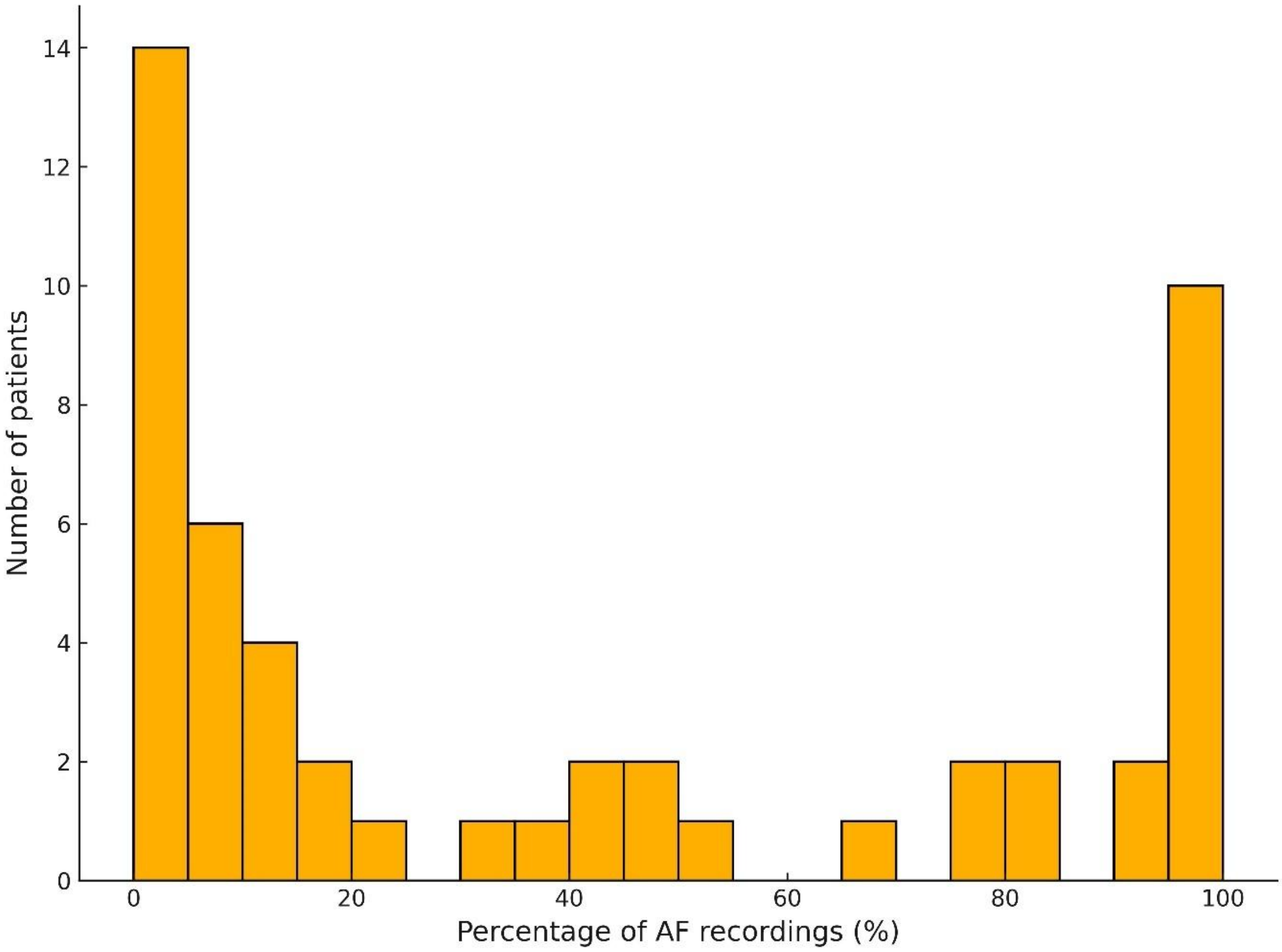

AF, Atrial Fibrillation

Supplementary Figure 8. Proportion of recordings with AF for patients with newly diagnosed AF stratified by FIND-AF 2.0 risk.

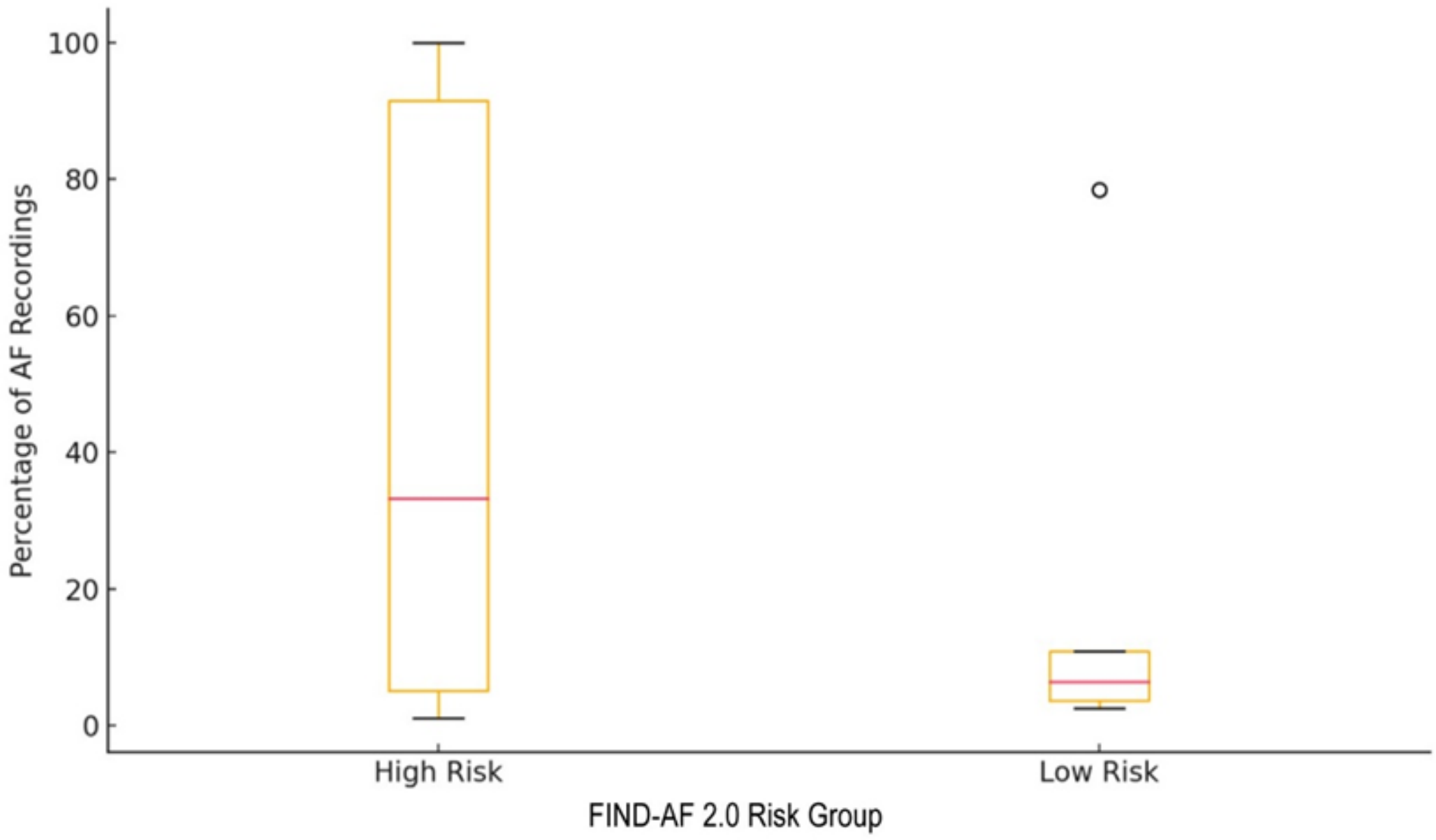

Supplementary Figure 9: Estimated AF diagnostic yield from intermittent ECG monitoring across FIND-AF 2.0 risk scores

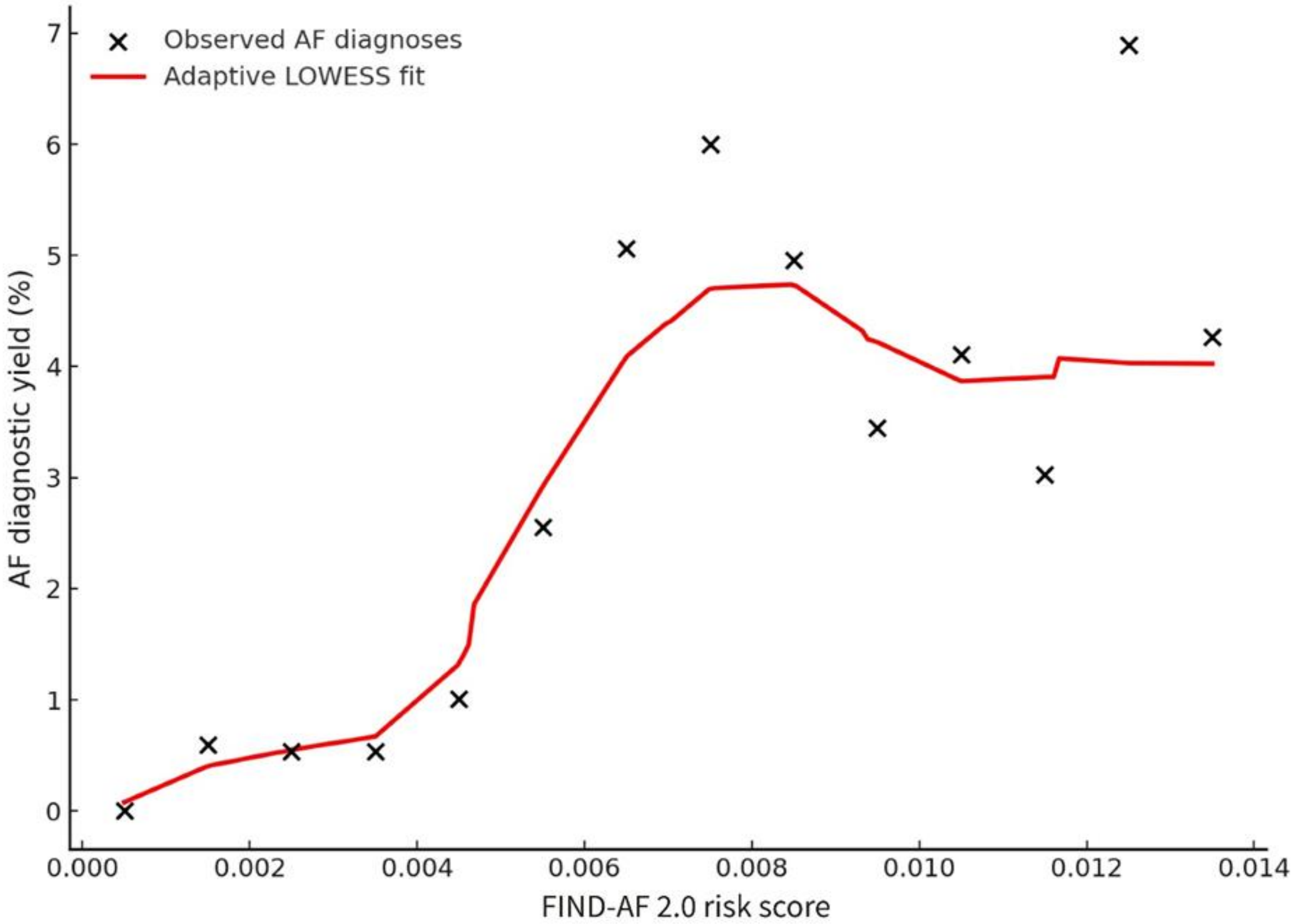

AF, Atrial Fibrillation; ECG, Electrocardiogram

Supplementary Figure 10: Cumulative incidence of ischaemic stroke within 12 months for patients with atrial fibrillation not anticoagulated in the FinACAF study stratified by FIND-AF2.0 risk and CHA_2DS_2-VASc

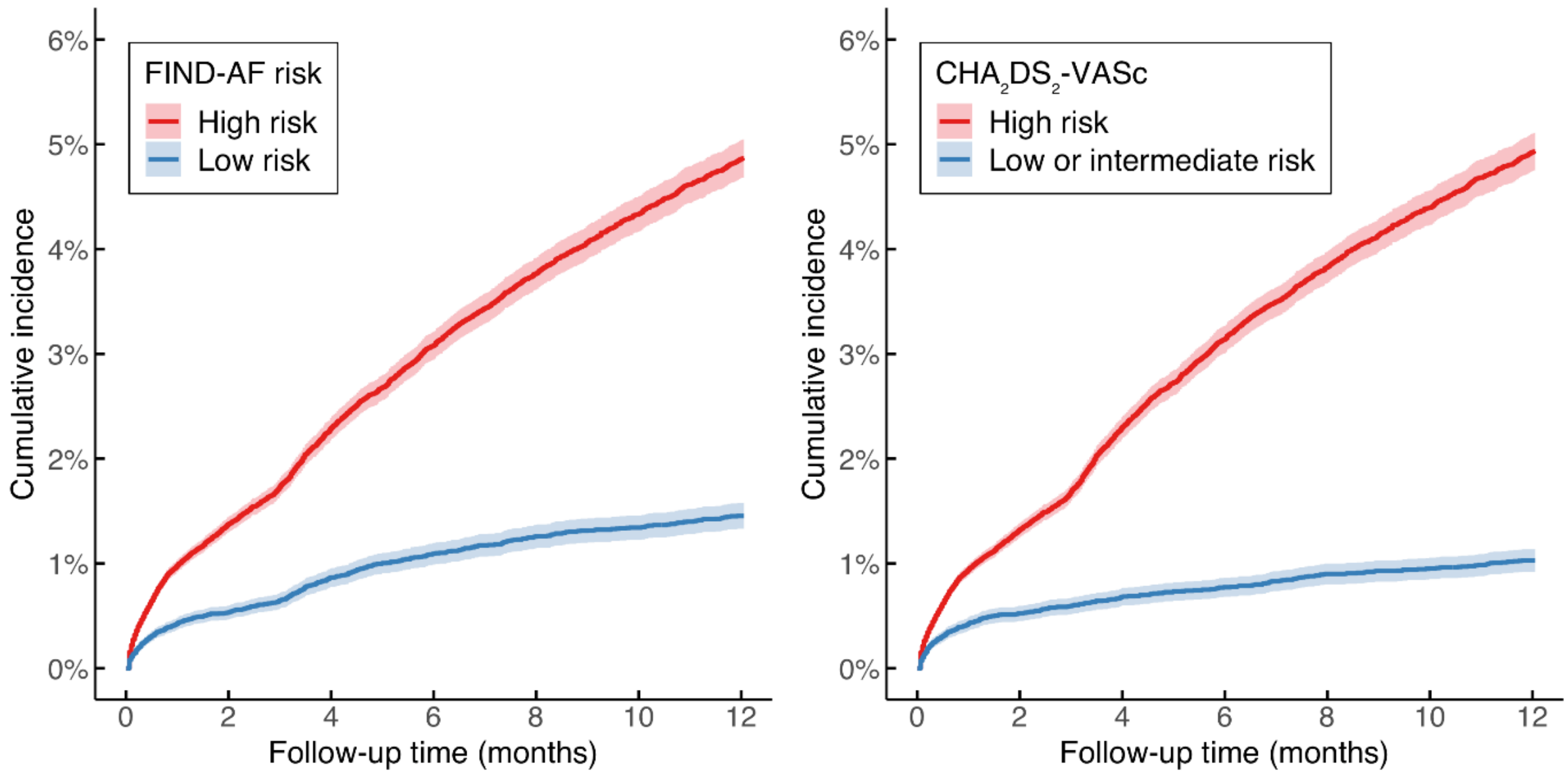

Supplement: Supplementary file 1 [file cir-154-707-s001.pdf]
